# A Capsid-Encoded PPxY-Motif Facilitates Adenovirus Entry

**DOI:** 10.1371/journal.ppat.1000808

**Published:** 2010-03-19

**Authors:** Harald Wodrich, Daniel Henaff, Baptist Jammart, Carolina Segura-Morales, Sigrid Seelmeir, Olivier Coux, Zsolt Ruzsics, Christopher M. Wiethoff, Eric J. Kremer

**Affiliations:** 1 Institut Génétique Moléculaire de Montpellier, Montpellier, France; 2 Université Montpellier I & II, Montpellier, France; 3 Max von Pettenkofer Institute, Gene Center, LMU Muenchen, Muenchen, Germany; 4 Centre de Recherche en Biochimie Macromoléculaire, Montpellier, France; 5 Department of Microbiology and Immunology, Loyola University Medical Center, Maywood, Illinois, United States of America; University of Pennsylvania, United States of America

## Abstract

Viruses use cellular machinery to enter and infect cells. In this study we address the cell entry mechanisms of nonenveloped adenoviruses (Ads). We show that protein VI, an internal capsid protein, is rapidly exposed after cell surface attachment and internalization and remains partially associated with the capsid during intracellular transport. We found that a PPxY motif within protein VI recruits Nedd4 E3 ubiquitin ligases to bind and ubiquitylate protein VI. We further show that this PPxY motif is involved in rapid, microtubule-dependent intracellular movement of protein VI. Ads with a mutated PPxY motif can efficiently escape endosomes but are defective in microtubule-dependent trafficking toward the nucleus. Likewise, depletion of Nedd4 ligases attenuates nuclear accumulation of incoming Ad particles and infection. Our data provide the first evidence that virus-encoded PPxY motifs are required during virus entry, which may be of significance for several other pathogens.

## Author Summary

Viruses exploit cellular functions during entry and exit of cells. To redirect cellular functions for their own purpose, viruses encode high-affinity binding sites for key-cellular factors. One such domain is the PPxY motif, which is present in structural proteins of several, mainly enveloped viruses. This motif binds to ubiquitin ligases of the Nedd4 family and recruits their function to sites of virus budding from cells. Here we show that adenoviruses also encode a PPxY motif in the internal structural protein VI and that the PPxY motif has an unprecedented function in virus entry. Adenoviruses with mutations in the protein VI PPxY motif undergo normal endosomal uptake and membrane penetration but have reduced infectivity, altered intracellular targeting and lack efficient gene-delivery. We also find that protein VI is ubiquitylated by Nedd4 ligases in a PPxY dependent manner following partial capsid disassembly and displays rapid intracellular movement. Depletion of Nedd4 ligases also alters virus movement within cells during entry and reduces viral infectivity. Given that PPxY motifs are important for virus exit our findings might have uncovered an additional function for PPxY motifs in virus entry, potentially expanding the significance of PPxY motifs and functionally related domains for viral replication.

## Introduction

Many viruses use the microtubule network of the host cell for transport to their site of replication (i.e. the nucleus) [1]. Access to the microtubule network is achieved through recruitment of cytoplasmic dynein motor proteins followed by efficient retrograde transport towards the nucleus [2],[3]. Virus-induced cellular signaling cascades help stimulate the directionality and efficacy of the transport [4]. Viral interaction with dynein motor proteins occurs either directly through capsid proteins or indirectly via hijacking of adapters from existing transport pathways [5]. Most DNA viruses accumulate transiently at the microtubule organizing center (MTOC) prior to nuclear translocation [1],[3],[6]. How they release from the microtubules or the MTOC and transport to nuclear pores is poorly understood. MTOC release may involve a switch from dynein to kinesin mediated transport, the cellular ubiquitin/proteasome system and/or nuclear transport receptors [1], [3], [5]–[8].

Indirect evidence that the host's ubiquitylation machinery participates in parts of the viral entry process comes from studies using pharmacological inhibitors of the ubiquitin/proteasome system. For example, translocation of a murine coronavirus from the endosome to the cytoplasm is facilitated by the ubiquitin-proteasome system [9]. Similarly, influenza viruses appear to be trapped in an endosomal compartment upon pharmacological inhibition of the proteasome [10]. In contrast, blocking the proteasome increases the transduction efficiency of adeno-associated virus vectors and this correlates with ubiquitylation of capsid proteins [11],[12]. The Semliki forest and the vesicular stomatitis virus, however, do not seem to be affected by proteasome inhibition during their entry suggesting different host factor requirements [10].

A role for the ubiquitylation machinery during egress of enveloped viruses is better understood. Egress involves the transport of assembled capsids, subviral structures or individual capsid proteins to assembly and budding sites at the cell surface or at intracellular membranes [1]. Budding, and potentially trafficking, to the egress site requires an intact class E vesicular sorting pathway (VSP, [13],[14]. The VSP is believed to involve the consecutive activity of three distinct heteromeric complexes termed endosomal sorting complexes required for transport (ESCRT-I, -II and –III, [15]). The capsid proteins of several enveloped viruses encode ‘late domains’ that specifically interact with ESCRT components and redirect them towards the site of viral egress [13]. Some late domains of the PPxY motif type (where x can be any amino acid) require the binding of ubiquitin ligases of the Nedd4 family of HECT-E3 ubiquitin ligases (Homologous to E6-AP Carboxyl Terminus) for efficient ESCRT recruitment [13].

Nedd4.1 and its close relative Nedd4.2 are prototypic members of this family, which is conserved from yeast to mammals [16]. They encode a N-terminal C2 domain for Ca^2+^-dependent lipid interaction, a catalytical HECT domain and three to four WW-domains for protein-protein interactions with proline-rich domains such as the PPxY motif [16],[17]. The exact role of PPxY-recruitment of Nedd4 ligases in VSP-mediated viral budding is still unclear. A possible link was recently shown by enhanced ESCRT ubiquitylation through Nedd4.2 overexpression [18],[19].

Late domains, including the PPxY type, have also been found in some nonenveloped reoviruses but a general function in virus release remains to be shown [20]. PPxY type late domains where also described for the Ad capsid protein penton, which can interact with ubiquitin ligases of the Nedd4 family. However, its role in Ad infection is unclear [21].

At least *in vitro*, Ad infects cells by first attaching to primary receptors, including CAR, CD46 and sialic acid, via the fiber protein [22]. In some cells endocytosis of Ad may be triggered by penton base-mediated signaling through alpha(v) integrins [23]–[25].

In epithelial cells, Ad serotype 5 (Ad5) particles undergo stepwise disassembly during entry, starting with detachment of the fiber at or near the cell surface and followed by a passage through endosomal compartments in which acidification serves as additional disassembly trigger for membrane penetration and cytosolic translocation [26],[27]. Partial disassembly releases the internal capsid protein VI, which can lyse membranes *in vitro* via its predicted N-terminal amphipathic helix [28]. In the cytosol, the particle engages in microtubule-directed transport towards the MTOC and is translocated to the nuclear pore complex (NPC) for nuclear import of the genome [29]–[31].

In this study we address the mechanisms of Ad cell entry. We demonstrate that the internal capsid protein VI is rapidly exposed to antibodies during cell entry, possibly at the cell surface or immediately after endocytosis. We further determine that protein VI remains partially associated with Ad capsids as they traffic to MTOCs and the NPC. We identify a functional PPxY motif within protein VI that mediates the association of protein VI with Nedd4 E3 ubiquitin ligases and facilitates its ubiquitylation. Recombinant Ad5 in which the protein VI PPxY motif is mutated have normal capsid morphology, escape from endosomes with similar efficiency as wildtype viruses, but are defective in genome delivery to the nucleus. We show that the PPxY motif in protein VI is involved in its efficient microtubule-mediated transport and mutating it in the virus alters the intracellular targeting of Ads towards the MTOC region concomitant with a post-entry block in viral infectivity. Furthermore, Nedd4.1 and Nedd4.2 are involved in Ad infection and intracellular targeting of incoming virions to the MTOC. We propose that the PPxY motif, in other viral systems, may also function during entry and interact with novel cellular pathways for efficient viral entry.

## Results

### Protein VI is exposed during Ad infection and partially remains associated with the viral particle

The fate of Ad particles immediately after internalization is only partially characterized. In the context of endosome escape of Ad5, very little is known about how this occurs and which, if any, cellular proteins are involved. From *in vitro* studies it was proposed that the internal capsid protein VI mediates Ad endosome escape [28]. Previous reports showed that protein VI dissociates from the Ad capsid very early after attachment [26],[27].

**Figure 1:**
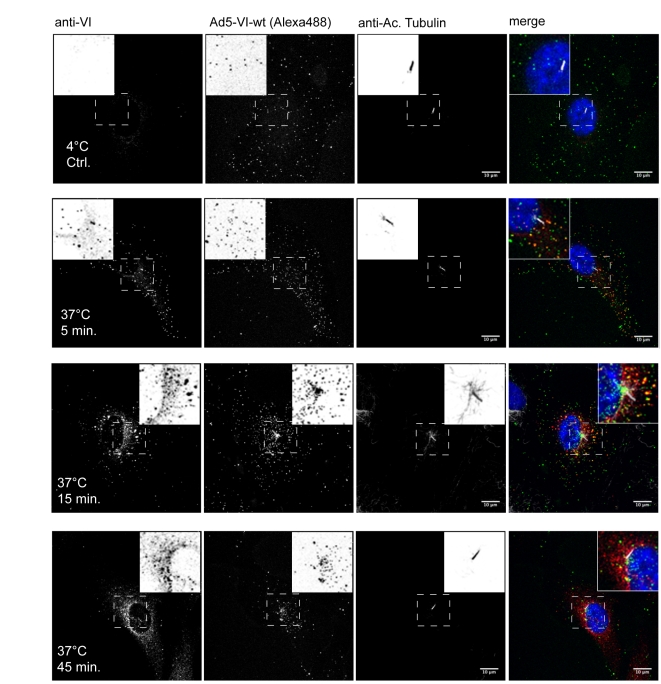
Timecourse of protein VI release during Ad entry. Ad5-488 was pre-bound to hTERT-RPE1 cells at 4°C (top row) and shifted to 37°C for 5 min (second row), 15 min (third row) and 45 min (bottom row) as indicated. Protein VI was detected using anti-protein VI antibodies (left column), adenoviral particles by detecting the Alexa-488 fluorescent signal (second column) and the MTOC by staining the primary cilia (third column). A composite of all three signals including the nucleus (in grayscale) is shown to the left. The inset shows an inverted magnification of representative virus, protein VI and the primary cilia signals from the small dashed box. In the composite protein VI signals are shown in red, Ad is shown in green, the primary cilia in grey and the nucleus in blue. Colocalization of protein VI and Ad appears as yellow. The scale bar is 10 µm.

To delineate the fate of protein VI during Ad entry we performed infection assays and followed the intracellular distribution of the viral capsid and protein VI as a function of time. To this end, fluorescently labeled Ad particles were adsorbed to either human retina epithelia pigment cells (hTERT-RPE1, Figure 1) or human osteosarcoma cells (U2OS, Figure S1) at 4°C and then transferred to 37°C to synchronize internalization. Cells were fixed at various times and analyzed by confocal microscopy (Figure 1). Protein VI was detected using an affinity purified polyclonal-antiserum and the location of the MTOC was marked by detecting the primary cilia, which originates at the MTOC using antibodies against acetylated tubulin [32]. At 4°C viral particles accumulated at the cell periphery showing sporadic positive staining with the protein VI antiserum (∼1%) possibly due to the recognition of protein VI from damaged particles (Figure 1 first row). In contrast, 5 min after the temperature shift, Ad particles were still localized close to the cell periphery but approximately 40% of them gave a signal with the protein VI antibody indicating that more protein VI was accessible (Figure 1, second row). After 15 min, particles had entered the cell with some localized at the MTOC region (Figure 1, third row) and some at the nuclear rim as described previously [6]. About 10% of the particles remained protein VI positive, including particles at the nuclear rim. After 45 min the majority of the particles were concentrated at the MTOC region (Figure 1, bottom row) as previously reported [3]. Protein VI staining was also concentrated at the MTOC region but most of the signal was not particle associated. Similar results were obtained in U2OS cells (Figure S1). Together these data suggested that structural rearrangements leading to protein VI exposure take place at or close to the cell surface during Ad entry. In addition, the data showed that protein VI trafficked to the MTOC region and partially remained associated with the capsid.

### Ad5 protein VI encodes a conserved PPxY motif determining viral infectivity

**Figure 2:**
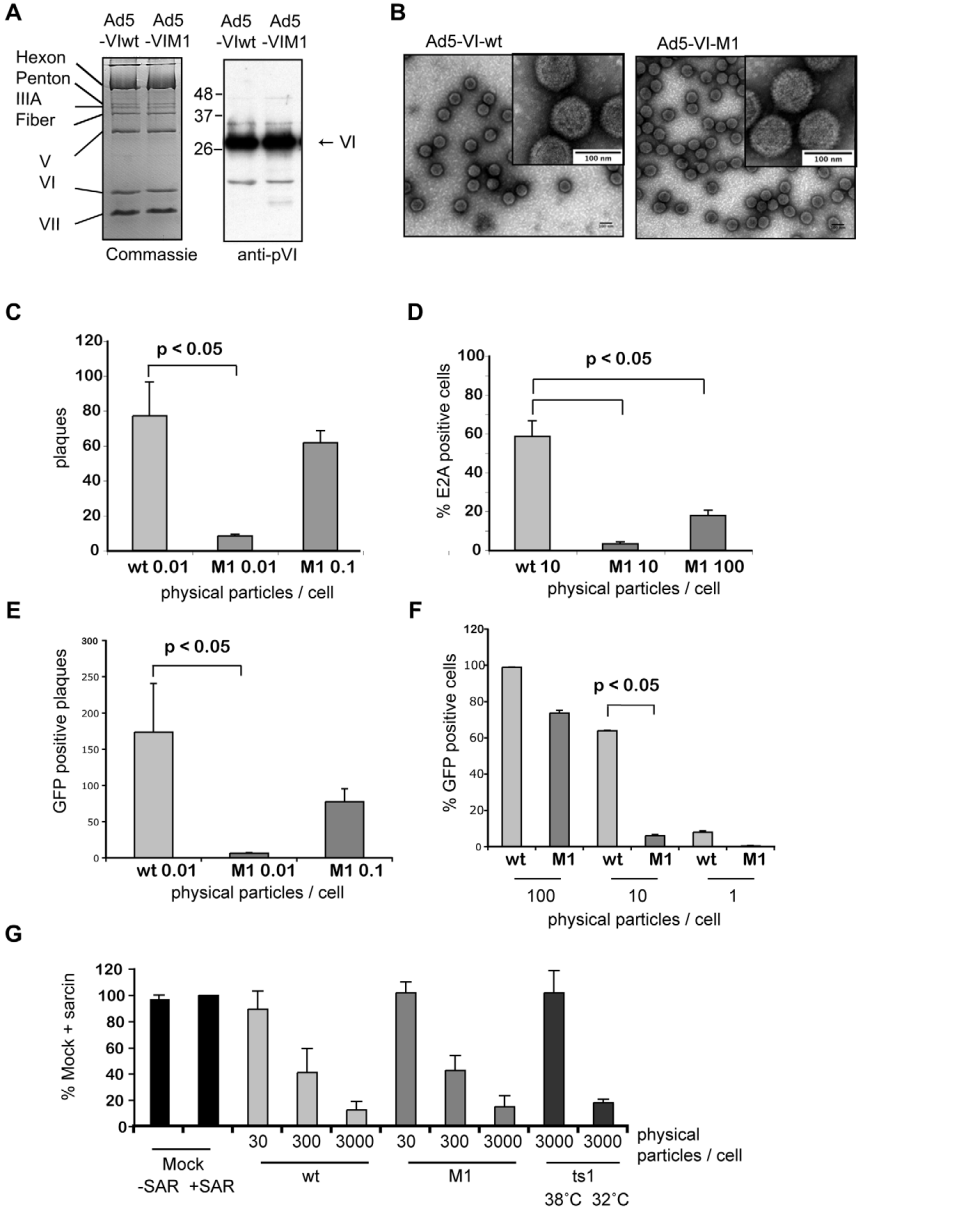
Reduced infectivity for an Ad with an altered PPxY motif. A) Biochemical comparison of Ad5-VI-wt vs. Ad5-VI-M1. Left panel; Coomassie gel comparison of wt Ad (Ad5-VI-wt, lane 1) and Ad with PPSY in protein VI mutated to PGAA (Ad5-VI-M1, lane 2). Individual capsid proteins are shown on the left. Right panel; western blot analysis of protein VI for Ad5-VI-wt (lane 1) and Ad5-VI-M1 (lane 2). B) Negative stain of purified Ad capsids. Electronic microscopy images of purified Ad5-VI-wt capsids (left panel) and purified Ad5-VI-M1 (right panel) are shown. The inset in each figure shows a magnification of individual capsids. The scale bar is 100nm. C) Plaque assay comparison of Ad5-VI-wt vs. Ad5-VI-M1. Quantification of plaques at day 12 for different physical particle per cell ratios for Ad5-VI-wt and Ad5-VI-M1. Shown is the average plaque number per 6 well (+/− standard deviation) of three individual experiments. D) Focus forming assay. E1 complementing 911 cells were infected with Ad5-VI-wt or Ad5-VI-M1 and replication centers were stained with E2A antibodies 24 h post-infection and at different particle per cell ratios. >5 random fields with >200 cells were counted, standard deviation represents field-to-field variations. E) Plaque forming assay comparison of Ad5GFP-VI-wt vs. Ad5GFP-VI-M1. Quantification of GFP positive plaques at day 12 at different physical particle to cell ratios. The values show the average number of GFP positive plaques per 6-well (+/− standard deviation) of two independent experiments. F) Single round infection assay comparison of Ad5GFP-VI-wt vs. Ad5GFP-VI-M1. U2OS cells were infected with increasing amounts of physical particle to cell ratios as indicated. GFP expression was quantified using FACS. Values correspond to the average percentage of GFP positive cells of two experiments done in triplicates (+/− standard deviation). Note that the ∼100% infection of wt infected cells at 100 physical particles per cell is saturated and not comparable to M1. G) Analysis of membrane penetration. A549 cells were infected with Ad5-VI-wt, Ad5-VI-M1, *Ad2ts1* grown at 32°C or at 38°C at different physical particle per cell ratios as indicated and in the presence of alpha-Sarcin and translational efficiency was determined by measuring the incorporation of radiolabeled amino acids over time. Values are given in percentage normalized to 100% translation measured in the presence of the toxin but in absence of virus. Conditions are indicated below each bar and are the mean of at least three independent experiments done in triplicates.

To identify possible trafficking determinants, we analyzed the sequences of protein VI from several Ad serotypes and identified a highly conserved ubiquitin-ligase interacting motif present in PPxY-type viral late domains (PPxY, Figure S2). To examine the role of this PPxY motif in Ad cell entry, we used an E1/E3-deleted Ad5 that had the protein VI PPSY motif mutated to PGAA (Ad5-VI-M1, detailed in Figure S3) [33],[34]. This mutation, when introduced into Mason-Pfizer monkey virus, was previously shown to abolish late domain functions with no apparent structural changes, which would impair virus assembly [35],[36]. To control for unintended mutations introduced during the cloning, we reverted the PGAA sequence back to PPSY (Ad5-VI-wt). Because the ∼360 copies of protein VI appears to be in contact with several proteins in the mature capsid, modifications that disrupt the tertiary structure could also affect the capsid composition. In large-scale preparations, mutant and wt virus banded at identical densities and gave similar yields of particles as determined by genome and protein quantification. A biochemical analysis of the capsid composition of purified viral particles showed no apparent differences between wt and mutant viruses (Figure 2A and data not shown). To confirm that viral capsid integrity between mutant and wt virus remained unchanged, we used negative stain electron microscopy. As shown in Figure 2B capsid integrity and morphology of the mutant virus was indistinguishable from the wt virus. In contrast, the infectious versus physical particle ratio of Ad5-VI-M1 was ∼20-fold lower than Ad5-VI-wt as assayed by plaque formation on monolayers of 911 cells (Figure 2C). Because infectious vs. physical particles can vary between preparations, we assayed plaque size, which is more informative measurement of propagation rate. Plaques were significantly smaller for Ad5-VI-M1 versus Ad5-VI-wt (see below), suggesting that the altered PPxY domain affects some stages of virus propagation.

To determine whether the M1 mutation influences Ad cell entry, we performed a fluorescent focus forming assay and stained cells at 8, 12 and 24 h post-infection for expression of the E2A protein, which marks the appearance of viral replication centers (Figure 2D and data not shown). Compared to Ad5-VI-wt, the Ad5-VI-M1 virus produced approximately 20-fold fewer fluorescent foci when equivalent numbers of viral particles were used for infections. This suggested that steps prior to replication (i.e. internalization) require an intact PPxY motif in protein VI.

To address trafficking using a different approach, we inserted a GFP expression cassette into the FRT site in the E1-deleted region of the wt and the M1 mutant virus (see Figure S3 for details). The GFP expressing viruses showed no difference in the quantitative yield and biochemical composition after large-scale purification. We repeated plaque forming assays (Figure 2E) and single round infection assays (Figure 2F), this time using GFP expression as the quantification method in non-complementing U2OS cells. We observed a reduction in infectivity in the same order of magnitude as previously (compare Figures 2C with 2E and 2D with 2F). In addition the GFP expression allowed us to follow the plaque formation over time. As shown in Figure S4 the spread of the M1 mutant virus was significantly slower and led to fewer and much smaller plaques (Figure 2E and Figure S4).

Next we asked whether Ad5 endosomal lysis efficiency following internalization is affected by the mutation in the PPxY motif of protein VI. We infected A549 cells with 30, 300 or 3000 particles per cell of either Ad5-VI-wt or Ad5-VI-M1 in the presence of alpha-sarcin, a membrane impermeable toxin that inhibits translation when it enters the cytoplasm. Alpha-sarcin enters the cell by virus-mediated endosomal membrane lysis thus providing a quantifiable marker for endsome membrane lysis (Figure 2G, [28]). We measured the incorporation of radiolabeled amino acids over time as a means to determine translational efficiency. As control we used the temperature sensitive Ad mutant *Ad2ts1* that is closely related to Ad5. *Ad2ts1* mutants poorly lyse the endosomal membrane when the virus is grown at non-permissive temperatures due to an increased capsid stability that lacks intra-endosomal disassembly. *Ad2ts1* mutants grown at permissive temperatures lyse the endosome with the same efficiency as the wt virus. Incorporation of radiolabeled amino acids was compared to alpha-sarcin treated cells. When we added alpha-Sarcin and Ad5-VI-wt, Ad5-VI-M1 or the *Ad2ts1* mutant grown at a permissive temperature translation was diminished in a dose-dependent manner over at least two orders of magnitude, showing efficient cytoplasmic delivery of the toxin. *Ad2ts1* grown at the non-permissive temperature did not inhibit translation in this assay (Figure 2G). We observed no difference between the PPxY-mutant virus and the wt controls. This indicated that an attenuating effect of the PPxY-mutation occurs after endosomal lysis and prior to the onset of replication.

**Figure 3:**
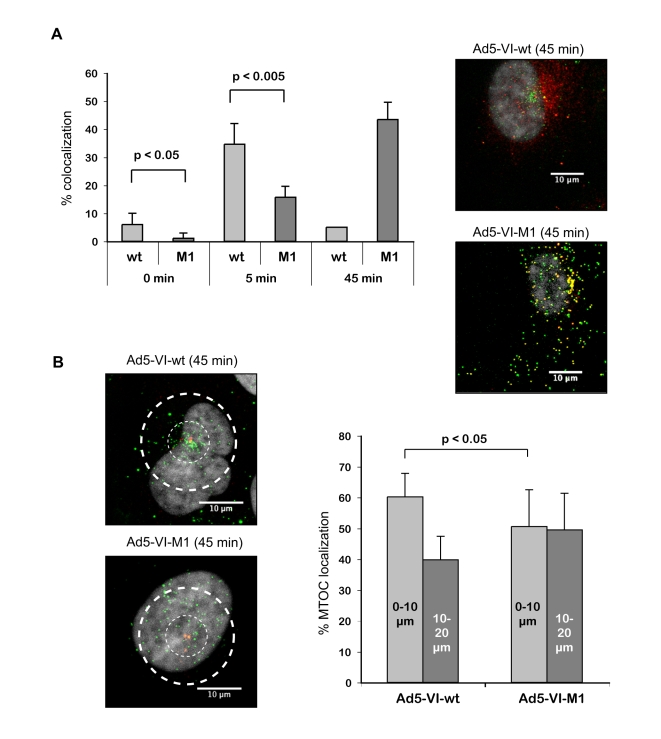
Adenovirus with altered PPxY motif lacks efficient protein VI release and shows reduced MTOC accumulation. A) Protein VI release from Ad5-VI-wt vs. Ad5-VI-M1 particles. Left panel; Shown is the percentage of viral particles that are positive for protein VI co-stain (+/− standard deviation) at different time points. Over 700 particles were counted for each condition. Due to the strong overlap of the signals 45 min after infection at the MTOC region for the wt, colocalization is given as an approximate value. Right panel; Intracellular localization of protein VI and capsids 45 min after infection. The panel shows Alexa488 labeled Ad5-VI-wt (top panel) or Ad5-VI-M1 (bottom panel) capsids in green and protein VI detected with protein VI specific antibodies in red 45 min after infection of U2OS cells. Colocalization of protein VI and the capsid appear as yellow. The scale bar is 10 µm. B) Ad-VI-M1 has a reduced MTOC accumulation. Left panel; Cells were infected with fluorescently labeled Ad5-VI-wt (top) or Ad5-VI-M1 (bottom). Subcellular localization in a representative cell is shown after 45 min. The scale bar is 10 µm. A quantification of the MTOC localization is shown to the right. Cells were synchronous infected with Alexa488 labeled Ad5-VI-wt or Ad5-VI-M1 respectively after preadsorption in the cold. 45 min post infection cells were fixed and stained for the MTOC using a rabbit anti-pericentrin antibody. To quantify the targeting of the virus towards the MTOC, confocal 0.4 µm sections were taken around the MTOC stain (∼3–5 sections), and combined using maximum image projection. Two concentric circles with 10 µm and 20 µm in diameter were positioned around the MTOC as shown in the left panel. Virus particles were then counted inside the 10 µm radius and in the region between 10–20 µm. The graph to the right shows the relative abundance within the two regions. Distribution for the wild type virus is shown on the left of the graph and for the PPxY mutated virus to the right. The analysis shows that mutated particles are less likely to accumulate at the MTOC then wt particles. The error bar represents cell-to-cell variation (n>15, p<0.05).

We next characterized the release of protein VI from fluorescently labeled Ad-VI-M1 and Ad5-VI-wt following synchronous infections of U2OS cells using the infection assay as described for Figure 1. Ad5-VI-wt released protein VI within 5 min (Figure 3A, left panel). In contrast, we observed a delayed release of protein VI from Ad5-VI-M1 (15% of M1 after 5 min compared to 38% of wt, Figure 3A) and an increase in colocalization of viral particles with protein VI at the nucleus (Figure 3A). This observation suggests a delayed accessibility of protein VI within the virus or a defect in protein VI dissociation from the virion (Figure 3A, images to the right). In addition to the increase of protein VI capsid association, Ad5-VI-M1 appeared to be more evenly distributed throughout the cell and did not efficiently accumulate at the MTOC region (Figure 3B, images to the left). Quantification revealed that 45 min post-infection approximately 60% of the wt viral particles in proximity of the MTOC could be found within a 10 µm radius around the MTOC and 40% within 10–20 µm. In contrast, the localization for the M1 virus was 50% for each region showing a decreased targeting towards the MTOC (Figure 3B, right panel). In summary these data suggest that the PPxY motif in protein VI is required for proper uncoating and normal nuclear targeting.

### Intracellular dynamics of protein VI depend on the PPxY motif and microtubules

**Figure 4:**
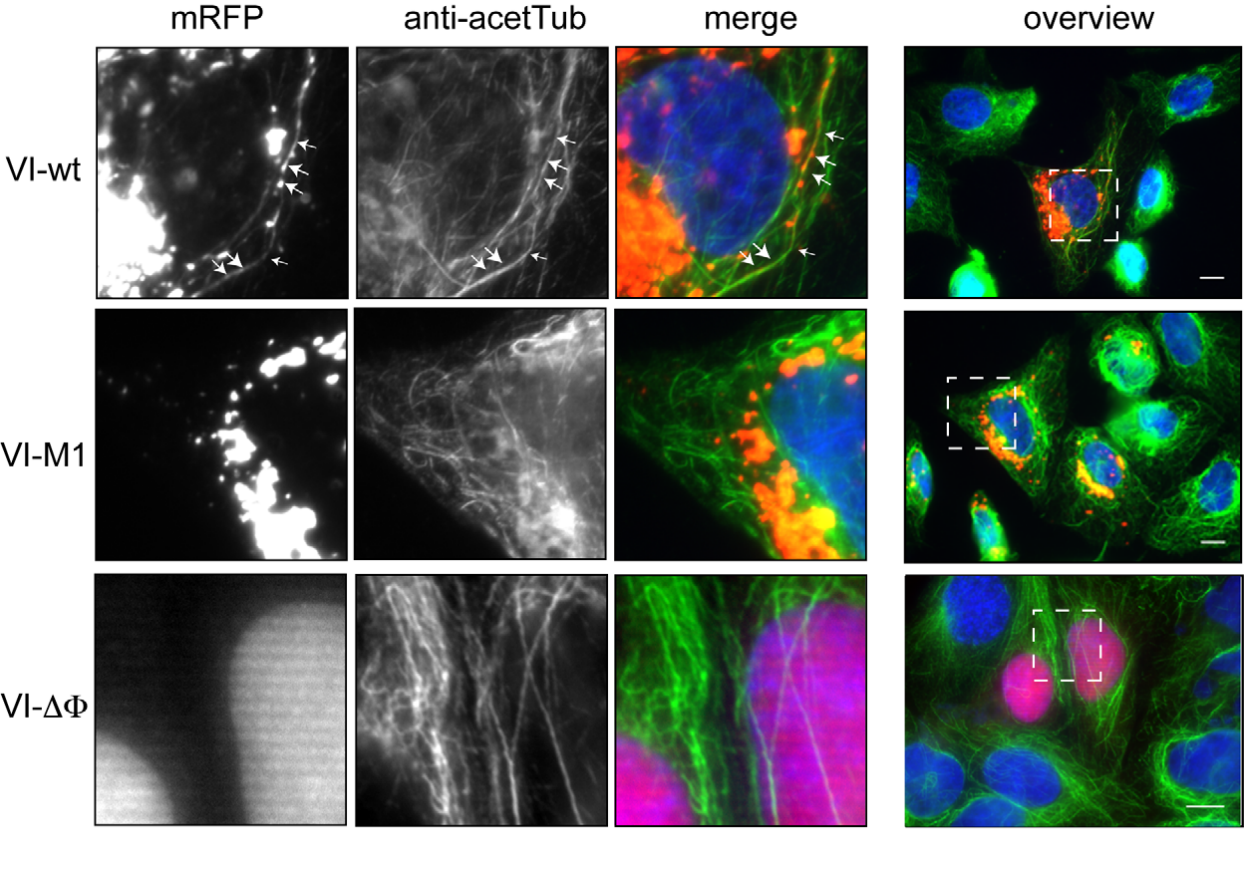
Subcellular localization of protein VI. U2OS cells were transfected with protein VI fused to mRFP, either using wt protein VI (VI-wt, top row), with mutated protein VI (VI-M1, middle row) or with deleted amphipathic helix (VI-ΔΦ, bottom row). Cells were co-stained for microtubules. The protein VI-signal is shown in the left column, the microtubule stain is shown in the second column and an overlay of the signals is shown in the third column. Protein VI fusions appear in red, microtubules in green and the nucleus in blue. The right column shows the field of cells from which the insets (small white square) was taken. The scale bar is 10 µm, Please note that the lower panel is a higher magnification. Association of protein VI with microtubules in tubulo-vesicular structures is indicated by arrows in the top row. Further examples of tubulo-vesicular structures can also be seen in Video S1 showing life-cell imaging of VI-wt transfected cells.

To understand the accumulation defect of Ad5-VI-M1 at the MTOC, we expressed wt (VI-wt), mutant (VI-M1) and protein VI deleted in the amphipathic helix (VI-ΔΦ) fused to mRFP in cells and analyzed protein VI localization in relation to microtubules (Figure 4). We found VI-wt in a punctuated distribution throughout the cell suggesting association with a vesicular compartment or tubulo-vesicular structures associated with microtubules (Figure 4, top row). In contrast, the PPxY motif mutant VI-M1 localized to a more central compartment and was rarely associated with the microtubules (Figure 4, middle row). Deletion of the amphipathic helix, in contrast, abrogated membrane association, causing nuclear targeting of protein VI and redistribution of protein VI from membrane fractions into soluble fractions ([37], and Figure 4 bottom row, data not shown).

**Figure 5:**
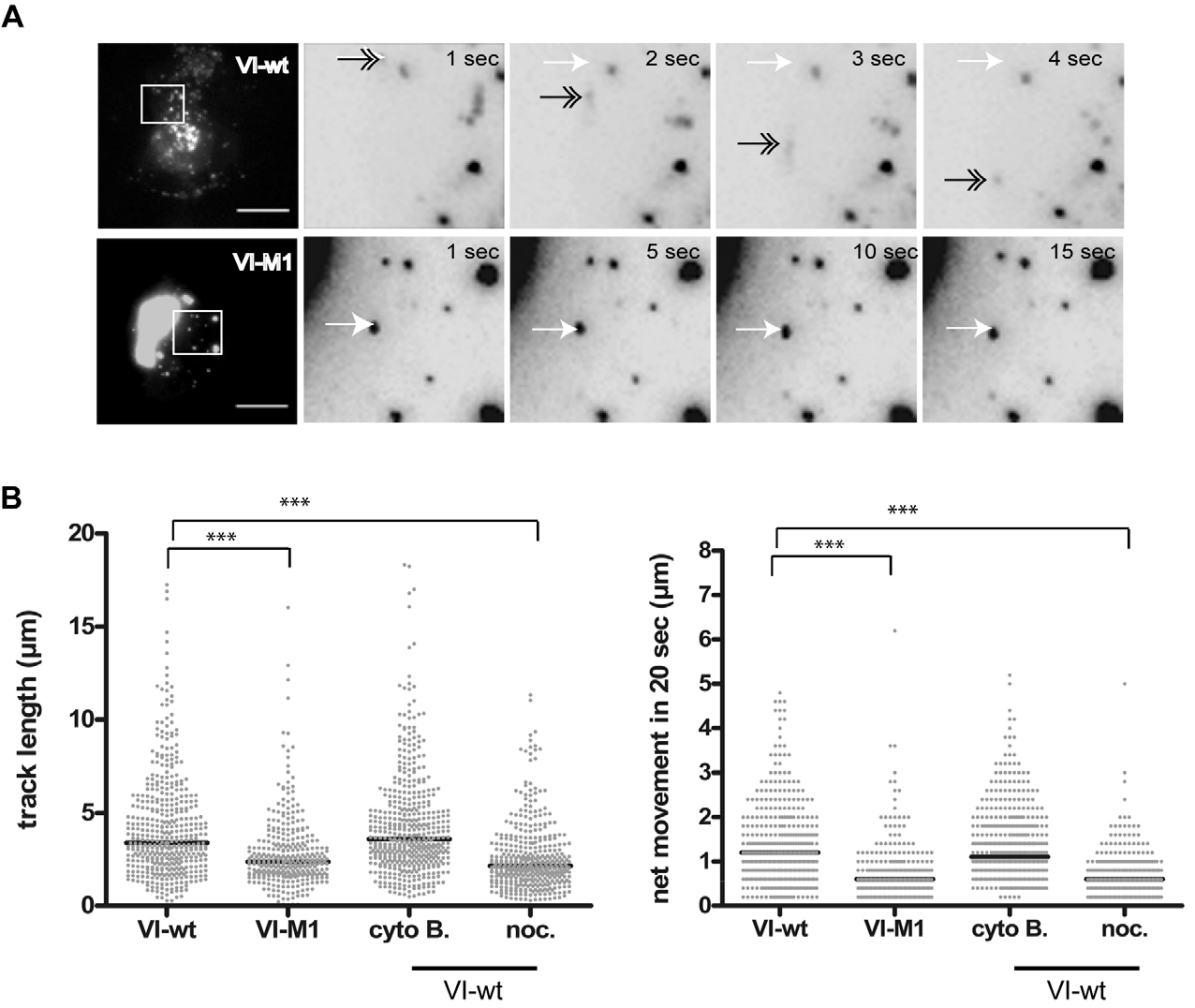
Subcellular dynamics of protein VI. A) U2OS cells were transfected with VI-wt (top row) or VI-M1 (bottom row) fused to mRFP and analyzed by live-cell imaging. Frames were taken at 1 sec intervals using a Cascade 512B 2 camera. The left image in all rows shows maximum image projections of the full frame depicting the cell with scale bar of 10 µm. The four inverted images to the right show magnifications from the boxed inset of four consecutive frames (in the case of VI-wt) and every fifths frame (in the case of VI-M1). In the top panel a moving particle is depicted by a grey arrow within each frame using the departure point depicted by a white arrow as reference. The lower panel shows VI-M1 not moving. B) Analysis of trajectory length and relative particle speed of VI-wt or VI-M1 (left side of both panels) or trajectory length and relative particle speed of VI-wt using drugs as indicated (right side of both panels). Movies were processed and all recorded trajectories were analyzed for length and relative speed using the Imaris™ software package. Individual particles were plotted for length of trajectories (in µm, left panel) and the net movement (in µm/20sec, right panel) under the conditions tested (indicated below each chart). Significant differences are indicated by bars.

Owing to its association with membranes, microtubules and the viral capsid, we next asked whether protein VI displayed intracellular dynamics that could explain virus trafficking. We therefore performed live-cell imaging (LCI) using cells expressing mRFP-VI-wt or mRFP-VI-M1. We found that VI-wt was fast moving with short- and long-range movements whereas VI-M1 was essentially motionless (Figure 5A and Video S1). The length of the trajectories and the movement of >300 particles were plotted (Figure 5B). We found that protein VI-M1 motility was greatly reduced compared to protein VI-wt. We next asked whether VI-wt motility depends on intact microtubules and/or actin filaments. Disrupting actin filaments with cytochalasin B had no apparent effect on VI-wt localization or motility (Figure 5B, right panel) suggesting that actin was not involved in the movement. In contrast, protein VI motility in nocodazole-treated cells was strongly reduced resembling the reduced motion observed for the M1 mutant (Figure 5B, right panel, see also Video S2 in the supporting information).

We asked whether VI-M1 showed only plus-end microtubule directed movement. We applied nocodazole to VI-M1 transfected cells, followed by washout of nocodazole. During treatment and after removal of nocodazole no movement or relocalization towards the cell center was observed for VI-M1. In contrast VI-wt rapidly stopped and restarted bidirectional movement under these conditions (data not shown). Together these data suggested that protein VI is a highly mobile protein that moves along microtubules, presumably in association with vesicular structures whose motion depends on the PPxY motif.

### PPxY motif is essential for protein VI ubiquitylation upon partial Ad disassembly

**Figure 6:**
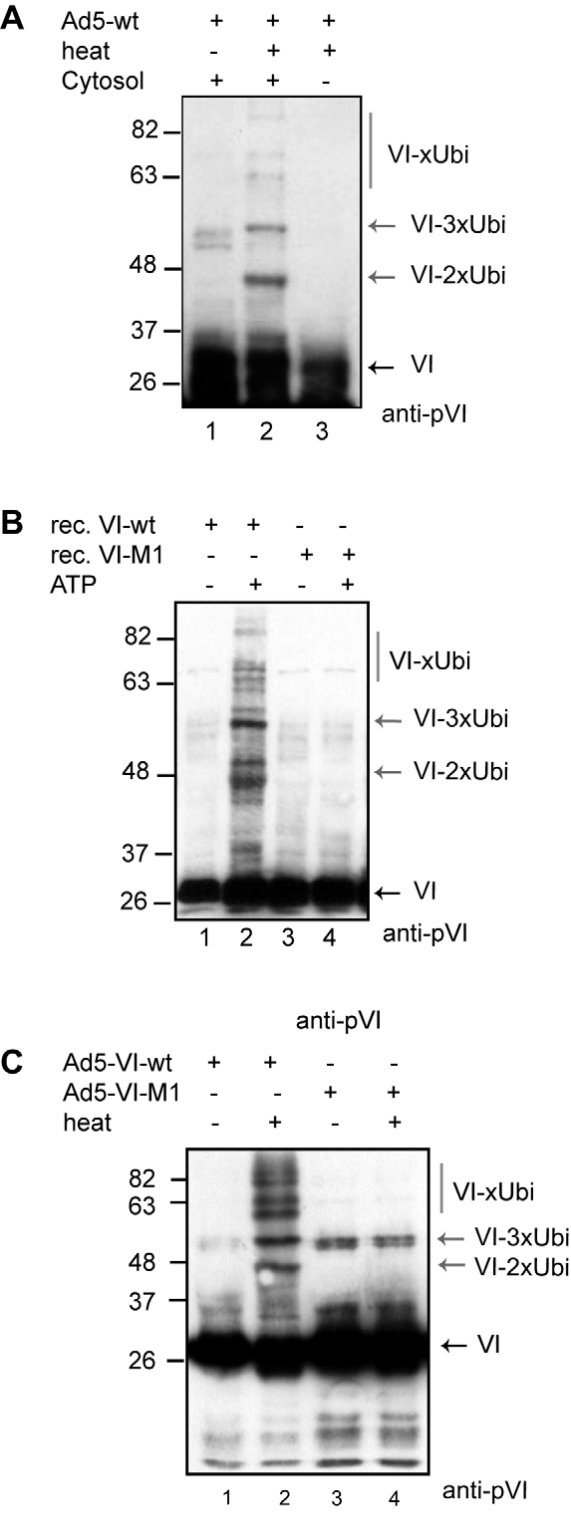
Protein VI is ubiquitylated following capsid disassembly. A) Purified Ad5 particles where used in *in vitro* ubiquitylation reactions, using ubiquitin, recombinant E1 and E2, an energy regenerating system and purified cytosol as source of the E3-ligase, and analyzed by western blot using antibodies against protein VI. Partial disassembly was induced by heat (lane 2 and 3). Cytosol was added to the reactions in lane 1 and lane 2. Protein VI-reactive bands are indicated by the black arrow and modified protein VI is indicated by grey arrows. B) Reactions as in A) using bacterially expressed protein VI-wt (lane 1 and 2) or VI-M1 (lane 3 and 4) with or without energy regenerating system as indicated (ATP). Western blot analysis using anti-protein VI antibodies is shown with ubiquitylated proteins marked with grey arrows (VI-Ubi) and unmodified protein VI (VI) is marked with a black arrow. C) Reactions as in A) followed by detection of protein VI by western blot using Ad5-VI-wt (lane 1 and 2) and Ad5-VI-M1 capsids (lane 3 and 4). Disassembly of capsids is indicated above each lane and protein VI reactive bands are marked as in B to the right.

PPxY domains are the physiological targets of ubiquitin ligases of the Nedd4 family [16]. Therefore we asked whether protein VI ubiquitylation depends on the PPxY motif. We adapted an *in vitro* Ad disassembly assay mimicking the partial capsid disassembly believed to occur during Ad entry by exposing virions to 48°C. This assay dissociates the vertices including fiber, penton, protein VI and peripentonal hexons, but leaves the remainder of the capsid intact (Figure S5; [28]). Heat and mock-treated samples were subjected to *in vitro* ubiquitylation reactions, using free ubiquitin, recombinant E1 and E2 enzymes and purified cytosol as source for the E3 ubiquitin ligase(s), and analyzed by western blot (Figure 6, [28]). Western blot analysis showed that partial capsid disassembly resulted in the appearance of protein VI reactive signals with discrete size increments suggesting predominant modification with two to three ubiquitin as well as some higher molecular weight bands (lane 2, Figure 6A). In contrast, the lack of capsid disassembly (lane 1) or cytosol (lane 3) showed no additional protein VI reactive bands. We also tested ubiquitylation of the capsid proteins fiber, protein IIIA and penton base as internal control. We only detected ubiquitylation of the penton base (which also harbors two PPxY domains at its N-terminus), while the fiber and protein IIIA (which lack PPxY motifs) were not modified (Figure S5). The ubiquitylation of protein VI was also confirmed by using GST-ubiquitin in the above assay, followed by GST-pulldown to show covalent modification of protein VI with ubiquitin confirming the predominant modification with two to three ubiquitin-moieties (data not shown).

To address the role of the PPxY motif in protein VI ubiquitylation, we repeated the *in vitro* ubiquitylation assay using wt or M1 mutant protein VI purified from *E. coli* followed by western blot analysis. We detected protein VI-reactive bands, consistent with protein VI modified with two to three ubiquitin (Figure 6B, lane 2). In contrast, no modification was observed when the PPxY motif was mutated (Figure 6B, lane 1) or in the absence of ATP (Figure 6B, lane 3 and 4). Using viral particles in *in vitro* ubiquitylation reactions (following partial capsid disassembly) protein VI of Ad5-VI-M1 was not ubiquitylated (Figure 6C, lane 3 and 4), while protein VI from Ad5-VI-wt was (Figure 6C, lane 1 and 2). Thus, the PPxY motif in protein VI is inaccessible in intact capsids but can recruit ubiquitin ligase activity from cytosol when protein VI is released from the capsid interior. Together our results show that protein VI ubiquitylation depends on i) virus disassembly, ii) an intact PPxY domain and iii) the presence of a cytosolic ubiquitylation activity.

### Protein VI binds to Nedd4 ligases via the PPxY motif

**Figure 7:**
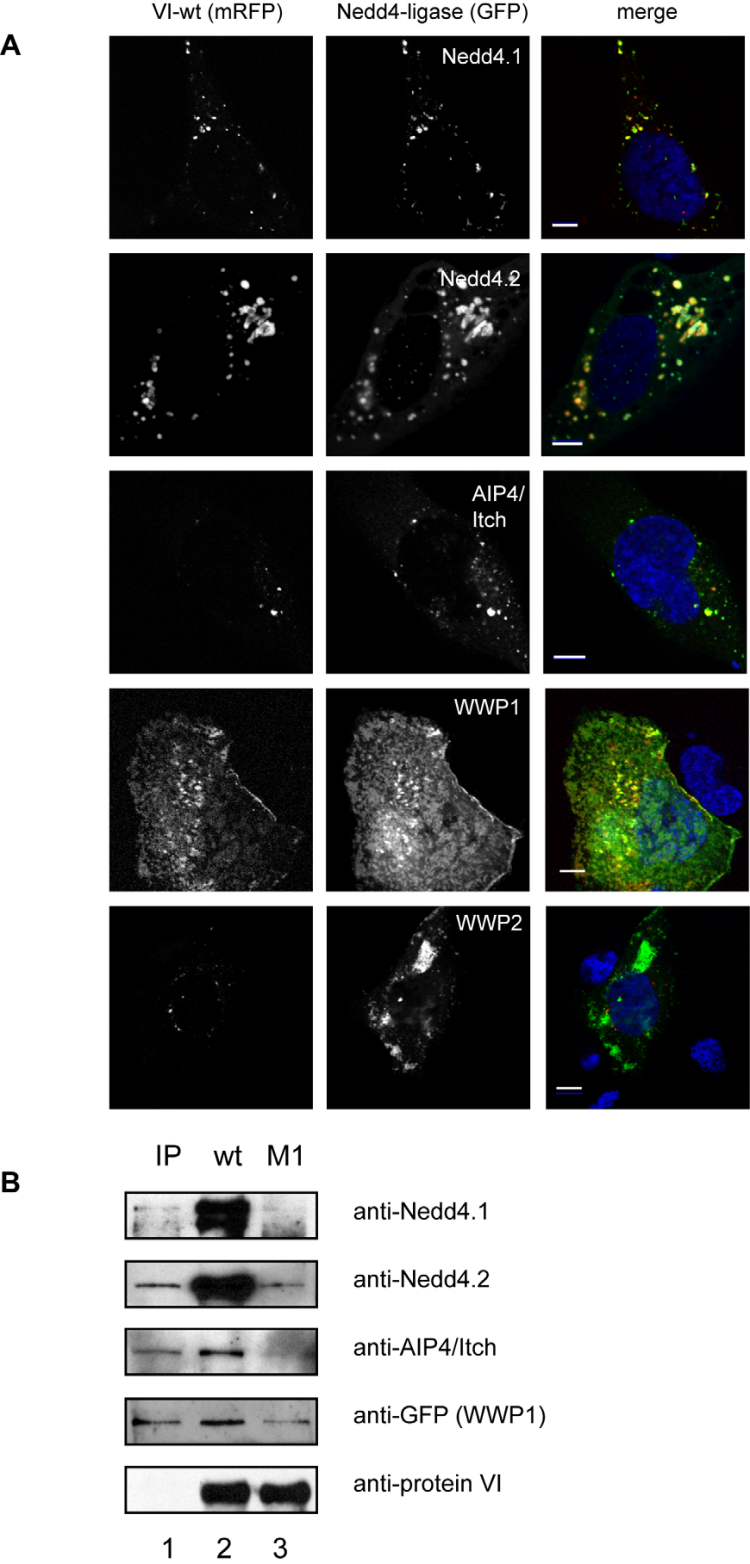
Protein VI interacts with Nedd4 ligases via the PPxY motif. A) Protein VI (VI-wt) was N-terminally fused to mRFP and co-transfected with GFP-Nedd4.1 (first row), GFP-Nedd4.2 (second row), GFP-AIP4/Itch (third row), GFP-WWP1 (fourth row) and GFP-WWP2 (last row). Confocal images of representative cells are shown and the mRFP signal for protein VI (left column), the GFP signal for the ligases (center column) and the merged signals together with DAPI stain of the nucleus (right column) is indicated above each column. Transfected plasmids are indicated. Colocalization of Nedd4 and protein VI results in a yellow signal. The scale bar is 10µm. B) Diverse GFP-tagged Nedd4 ligases were over-expressed in cells and cytosolic extracts were used for pulldown experiments using recombinant protein VI-wt or VI-M1 coupled to beads. 10% of the input material (IP) is shown in the first lane. Bound material for protein VI-wt (lane 2) and VI-M1 (lane 3) was detected with respective antibodies as indicated to the right. Co-eluted protein VI detected with a protein VI specific antibody is shown as loading control in the lower lane.

To identify the ligase responsible for protein VI ubiquitylation, we focused on the Nedd4-family members Nedd4.1, Nedd4.2, AIP4/Itch, WWP1 and WWP2 because they can interact with viral late domains that harbor PPxY motifs [38]. We first co-expressed the VI-wt or VI-M1 mRFP fusion protein together with each of the E3 ligases fused to GFP in U2OS cells. When expressed alone, most ligases localized primarily to the cytoplasm (data not shown, WWP1 localized to the plasma membrane and WWP2 accumulated in an uncharacterized intracellular membrane compartment). In contrast, when VI-wt is coexpressed with Nedd4.1, Nedd4.2 or AIP4/Itch, the ligases are recruited to the same membrane compartment as protein VI (Figure 7A, row 1–3). WWP1 appears to sequester protein VI at the plasma membrane (Figure 7A, row 4). WWP2 does not colocalize with VI-wt (Figure 7A, row 5). We did not detect significant colocalization between VI-M1 and the E3 ligases, consistent with a PPxY-dependent interaction (Figure S6).

To determine whether any of the ligases specifically interact with protein VI, we used purified cytosol from cells overexpressing GFP-tagged ligases and performed pull-downs with beads coated with recombinant protein VI-wt or VI-M1. Two ligases, Nedd4.1 and Nedd4.2, were highly enriched on VI-wt beads while none of the other ligases showed strong binding to VI-wt- or to VI-M1-beads (Figure 7B). Taken together, these data suggest a preferential interaction between Nedd4.1 and Nedd4.2 and the PPxY motif in protein VI, which leads to relocalization of the ligases from the cytoplasm to a membrane compartment.

### Nedd4 ligases ubiquitylate protein VI via the PPxY and reduce Ad transduction and MTOC accumulation

**Figure 8:**
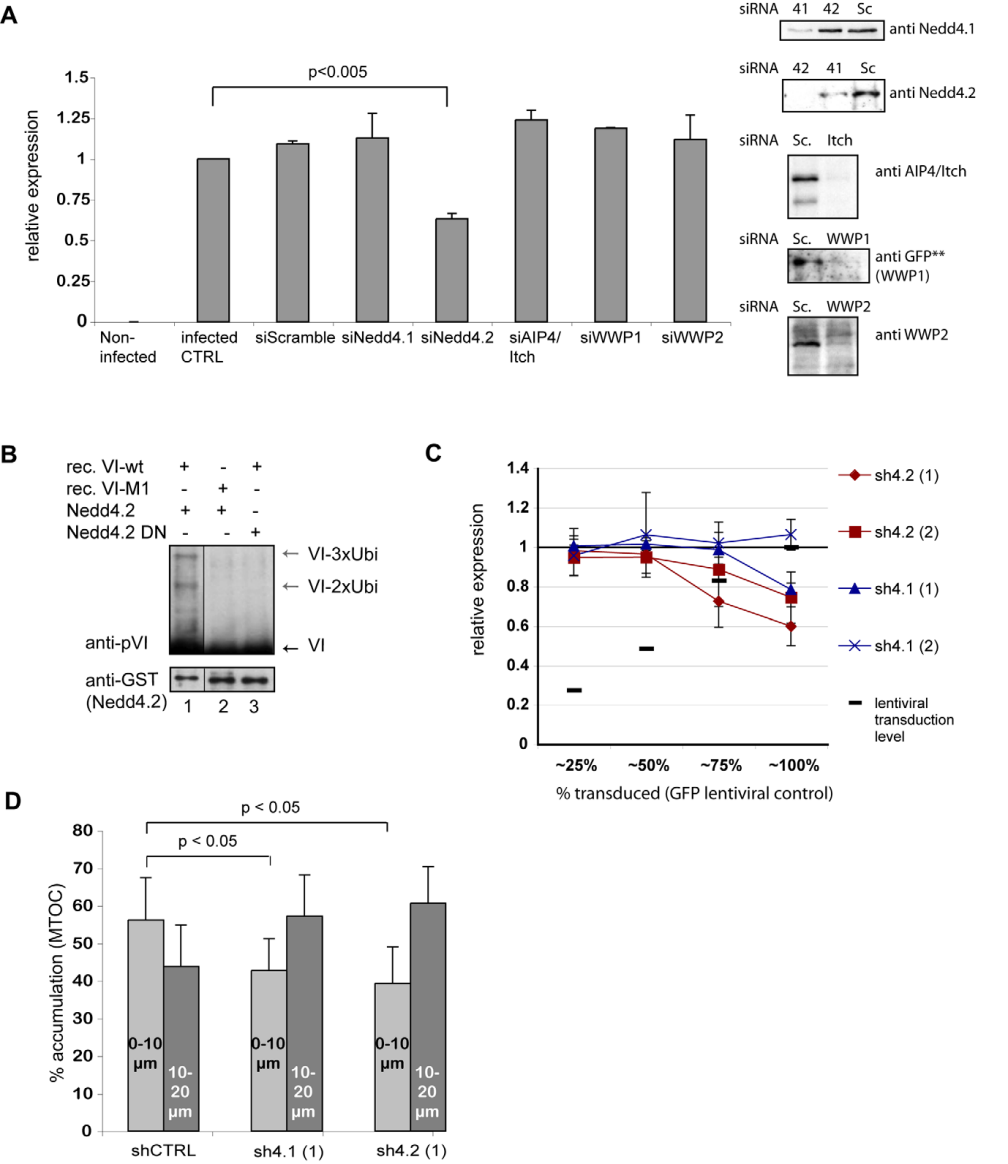
Nedd4.2 ubiquitylates protein VI and is required for efficient Ad transduction and MTOC accumulation. A) Effects of Nedd4 depletion on Ad transduction. U2OS cells were transfected with siRNAs specific for Nedd4.1, Nedd4.2, AIP4/Itch, WWP1 and WWP2 or scrambled siRNAs. Western blot controls for depletion are shown to the left (except for WWP1 where depletion of transfected WWP1-GFP was detected using GFP specific antibodies because no specific antibodies were available for detection of the endogenous protein). Following depletion, cells were transduced with AdGFP. Relative GFP expression is shown. Statistical analysis was performed using pairwise comparison using the Mann & Whitney-test showing significant reduction for Nedd4.2 depleted cells. B) *In vitro* ubiquitylation reaction using recombinant active Nedd4.2 (lane 1 and 2) or inactive Nedd4.2 (lane 3). Recombinant VI-wt (lane 1 and 3) or VI-M1 (lane 2) was used as substrate. Modified protein VI (grey arrows) or unmodified protein VI (black arrows) was detected following western blot analysis. Antibodies are indicated to the left of each blot. C) Dose dependent depletion of Nedd4.2 and Nedd4.1. U2OS cells were transduced with increasing amounts of shRNA expressing lentiviral vectors achieving transduction levels as indicated (from 25–100%) and transduced with AdGFP. GFP expression levels were determined 24 h later using flow cytometry. GFP expression levels within each condition were normalized to transduction controls of cells treated with shRNA expressing lentiviral vectors against luciferase (arbitrarily set to 1). Values are the mean (+/− standard deviation) of at least two experiments done in triplicates D) Accumulation of Ad at the MTOC region following Nedd4 depletion. Fluorescently labeled Ad was used to infect cells following control depletions (shLuc) or depletion of Nedd4.1 or Nedd4.2 using (sh4.1 (1) and sh4.2 (1) as in C). Subcellular localization of viral particles was determined at 45 min. post infections. Cells were fixed and stained for the MTOC using a pericentrin antibody. Particles were counted and scored for their relative proximity to the MTOC using two concentric circles with 10 and 20 µm diameter around the MTOC (compare also Figure 3). The graph shows percentage of viral particles within 10µm or 10–20 µm proximity to the MTOC as indicated. Note that in Nedd4.1 and Nedd4.2 depleted cells the relation is inverted compared to control depleted cells showing less viral particles accumulating at the MTOC. The error bar represents cell-to-cell variation (n>15, p<0.05).

To further characterize the interaction between protein VI and the ligases, we knocked down Nedd4.1, Nedd4.2, AIP4/Itch, WWP1 and WWP2 using siRNAs (Figure 8A). The cells were then incubated with an Ad5 vector harboring a GFP expression cassette (AdGFP) at a low multiplicity of infection (30 physical particles per cell) for 3 h to achieve approximately 20% transduced cells and limit the time of virus exposure. The following day the percentage of GFP-positive cells was quantified by flow cytometry. Most ligase knockdowns had no significant effects on transduction, but Nedd4.2 knockdown diminished transduction by 50% (Figure 8A).

Because Nedd4.2 showed the strongest effect on Ad transduction we determined whether bacterially expressed and purified Nedd4.2 could ubiquitylate purified protein VI *in vitro*. A minimal system where cytosol was replaced by recombinant Nedd4.2 was sufficient for protein VI ubiquitylation (Figure 8B, lane 1). The ubiquitylation pattern was similar to that obtained with cytosol (Figure 6) and required an intact PPxY and a catalytically active Nedd4.2 (Figure 8B, lane 2 and 3).

Nedd4.1 and Nedd4.2 both showed strong interaction with protein VI in pulldown assays. To further investigate the role of each ligase we transduced cells with lentiviral vector expressing shRNAs against either Nedd4.1 or Nedd4.2 or luciferase as a control. We transduced cells in a dose-dependent manner to achieve different levels of knockdown. Transduction efficiency was monitored using a GFP expressing lentiviral vector in control cells. Seven days post-transduction shRNA treated cells were infected with AdGFP virus as described above and the transduction rate was determined by flow cytometry. We observed a dose-dependent decrease in infectivity for two different shRNAs against Nedd4.2, which was similar to what we observed when we used siRNAs (Figure 8C). The results for shRNAs against Nedd4.1 were less clear. One shRNA also reduced viral infection at very high transduction rates but to a lesser extent than shRNAs against Nedd4.2 while a second shRNA showed no effect on Ad transduction (Figure 8C). A combined treatment of cells with either siRNAs or shRNAs against Nedd4.1 and Nedd4.2 did not further decrease Ad-transduction indicating that the effects of Nedd4-ligase knockdowns on Ad transduction may be complex (data not shown).

A hallmark of Ad5 infection is its transient accumulation at the MTOC during entry [5]. The mutation of the PPxY in the Ad5-VI-M1 virus seemed to alter this localization (Figure 3). Similarly, the PPxY motif was required for Nedd4.2 dependent ubiquitylation of protein VI. Because knockdown of Nedd4 ligases also diminished transduction with AdGFP vectors we examined accumulation at the MTOC region in cells depleted with control shRNA and Nedd4.1 and Nedd4.2 specific shRNAs. We used the same strategy as in Figure 3 by quantifying viral particles in proximity to the MTOC, which was identified by stain for pericentrin. MTOC accumulation for Nedd4.1 and Nedd4.2 shRNA treated cells was reduced when compared to cells treated with control shRNA indicating that both ligases might be involved in proper targeting of viruses towards the MTOC (Figure 8D).

In summary, these data provide evidence that release of protein VI during entry and a possible interaction between the PPxY motif of protein VI and Nedd4-family ligases are determinants of Ad5 trafficking during infection.

## Discussion

In this study we show that the Ads internal capsid protein VI harbors a PPxY-motif that is involved in virus entry and infectivity. For Ads, reaching the nucleus requires a series of sequential steps: receptor-mediated endocytic uptake, partial capsid disassembly, endosomal rupture, microtubule based transport to the MTOC and nuclear trafficking. The link between these steps has been best exemplified in the case of the thermostable temperature-sensitive mutant *Ad2ts1*. This mutant enters cells by receptor-mediated endocytosis, but remains in an endosomal compartment due to increased capsid stability. Therefore, *Ad2ts1* particles are directed to lysosomes for destruction and/or recycled back to the surface thus precluding accumulation at the nuclear pore complex [26],[28],[39]. The role of facilitating endosomal escape during Ad entry was initially assigned to the penton base [40]. Later, Wiethoff and co-workers showed that most membrane lytic activity of Ad viral capsids comes from the predicted N-terminal amphipathic helix of the internal capsid protein VI, and that membrane lytic activity required partial capsid disassembly to release protein VI [28].

Here we present several lines of evidence that protein VI plays an additional and previously unidentified role in nuclear targeting of the Ad capsid. We show that protein VI exposure from Ad5 capsids occurs within minutes when pre-adsorbed Ad5 is shifted from 4°C to 37°C. This is consistent with the loss of the fiber prior to internalization and the rapid accumulation of Ad5 in the cytosol [41],[42]. We found that significant amounts of protein VI remains partially associated with the viral capsid after the initial exposure and until the viral particle accumulates at the MTOC or the nuclear rim.

We show that protein VI is engaged in rapid intracellular trafficking that depends on intact microtubules and requires the N-terminal amphipathic helix for microtubule association and the PPxY motif for motion. To our knowledge protein VI is the first Ad capsid protein described that possesses its own microtubule-dependent dynamics and future work has to address if other capsid proteins have similar properties. Inactivation of the PPxY in the viral context (Ad5-VI-M1) resulted in a post-entry delay that reduced infectivity and prevented efficient accumulation of the entering virus particles at the MTOC. While we cannot exclude the possibility that the PPxY motif in protein VI also plays a role in adenoviral replication, assembly or egress, our single round infection assays showed that the majority of the titer reduction for the mutant was related to steps prior to the initiation of replication or the delivery and expression of a reporter gene. Moreover, our data show that the efficiency with which a toxin is delivered to the cytosol during Ad infection is similar for mutant and wt virus. This provides strong evidence that the PPSY motif in protein VI has a function during cell entry, but probably only after endosomal membrane lysis has occurred. Current structural data place protein VI inside the assembled capsid, therefore potentially precluding it from functions during egress at least when capsid associated [43]–[45]. Our observations show that protein VI is exposed after entry and that capsid disassembly is required for its ubiquitylation, which is consistent with our hypothesis that the PPxY motif is accessible only during Ad entry following partial capsid disassembly. Furthermore, it is currently not clear whether late domains containing PPxY motifs present in other viral systems, which are required for viral egress, have additional functions. Mutational inactivation of PPxY motifs in the VP40 structural protein of Ebola virus and matrix protein of rabies virus both showed an attenuation of the virus and a reduction of infectivity [20],[46]. Interestingly, for Ebola virus, the PPxY mutants showed no budding defect, but virus production was reduced, which could indicate a disruption earlier on in the life cycle than previously thought [46]. For rabies virus, Wirblich et al. describe a budding defect of the late-domain mutant but also noted a reduction of early mRNA production [20].

Earlier studies have shown that PPxY type late domains bind to ubiquitin ligases of the Nedd4 family rather then directly to class E VSP proteins [13]. Our study showed that the PPxY motif in protein VI can interact with all Nedd4 ligases tested but preferentially binds to Nedd4.1 and Nedd4.2 and re-targets them to membranes. Owing to topological constrains, recognition of the PPxY domain should require membrane rupture because of the cytosolic localization of Nedd4 ligases, which according to our data does not seem to be affected by the PPxY mutation in the M1 virus.

The PPxY motif in protein VI seems to favor interaction with Nedd4.1 and Nedd4.2 although we observed interactions with AIP4/Itch and WWP1 as well. It is possible that an interaction between protein VI and WWP1, as we observe in transient transfections, is circumvented by exposure of the PPxY domain after the virus has entered the endosomal compartment. A role for Nedd4.1 or Nedd4.2 in Ad entry is underscored by our observation that Nedd4.2 can directly ubiquitylate protein VI via the PPxY motif and its depletion (and to a lesser extent also depletion of Nedd4.1) reduces Ad transduction. In addition this depletion also reduced MTOC accumulation of viral particles following infection, which is similar to the effect of the PPxY mutation in the M1 virus. However the effects were modest, indicating that additional mechanisms contribute to Ad entry. Further studies will be needed to identify a specific role for each ligase in Ad entry and trafficking towards the MTOC and to determine whether other ligases like AIP4/Itch and WWP1 are involved.

How ubiquitylation of protein VI or interaction with Nedd4 ligases directs accumulation of Ads at MTOCs remains unknown. Ubiquitylated protein VI could be specifically recognized by cellular factors. Alternatively, recruitment of Nedd4.1 and/or Nedd4.2 by protein VI could result in the ubiquitylation of other cellular factors that constitute an efficient transport means used by the virus. Recent work has shown that some members of ESCRT-I become ubiquitylated when Nedd4.2 is overexpressed [18],[19]. Therefore, it is possible that Nedd4.2 (or other Nedd4-ligases) binding to protein VI could activate the ESCRT pathway via ubiquitylation. Whether membrane compartments or the ESCRT pathway plays a direct role in Ad virus transport during entry remains to be addressed. However, ESCRT components can be found at the endosomal compartments as well as associated with the centromeric region [47]. It is noteworthy that endosomal escape is also required for interferon induction by Ad via yet unknown mechanisms [48]. This pathway may also be related to protein interaction between protein VI and Nedd4-family ligases.

Release and separation of protein VI from the capsid is clearly defective in the Ad5-VI-M1 mutant virus. A lack of functional PPxY may therefore block disassembly steps, preclude efficient endosomal escape following the membrane lysis event or fail in the recruitment of subsequent factors required to efficiently link the virus with retrograde transport pathways. Penton base also encodes PPxY motifs [49]. Because eliminating the PPxY motif from protein VI had only modest effects on MTOC accumulation, there may be functional overlap between the PPxY in protein VI and penton base. This is further supported by the observation that ubiquitylation of penton could be abolished by depleting the cytosol with protein VI indicating that maybe the same ligases are involved (Figure S5).

In conclusion, our study is the first demonstration of a PPxY motif, previously described for viral late domains, present in a non enveloped DNA virus that exerts a function during entry. Conservation of the amphipathic helix and PPxY motif in all *Mastadenovirus* suggests that protein VI fulfils a key function. We suggest that after endocytosis cell or serotype specific capsid disassembly cues regulate exposure, membrane-interaction and ubiquitylation of protein VI (and possibly other capsid proteins). This could facilitate the recruitment of a common cellular microtubule-dependent pathway for retrograde trafficking. This mechanism could be more important *in vivo* in highly polarized epithelia or neurons where long-range movement is crucial and might be less important in cell culture models [50]. Given the prevalence of viral late domains, ubiquitylation and trafficking towards the MTOC our results may have uncovered a more general mechanism by which viruses and other cargos achieve intracellular delivery and provide a rational to look for further “early” functions of PPxY motif containing late-domains in other viral systems.

## Materials and Methods

### Cell lines, cell culture and virus production

Immunofluorescence experiments and infection experiments were performed using U2OS cells (human bone osteosarcoma epithelial cells), hTERT-RPE1 cells (human retina epithelia pigment cells, Clontech) or A549 cells (human alveolar basic cells). Cytoplasmic extracts for pulldowns were prepared from 293T cells (human embryonic kidney cells). All cells except hTERT-RPE were grown in DMEM Glutamax™ (Gibco) supplemented with 10% of fetal calf serum (FCS) (Biowest). hTERT-RPE1 cells were a kind gift from M. Bonhivers (University of Bordeaux 2) and grown in DMEM/HamsF12 media supplemented with 10% FCS according to the suppliers instructions. Prior to infection experiments, cells were serum starved for 24h to induce primary cilia growth [32]. Recombinant Ad5-VI-wt and Ad5-VI-M1 viruses and their GFP expressing counterparts were constructed as described in the supplemental material. Amplification of viruses was done in 293 cells and purified using double CsCl_2_-banding. Virus particle to cell ratios were calculated based on the estimated copy numbers of viral genomes. Copy numbers were calculated according to Mittereder et al. [51]. Briefly, purified particles were diluted 1∶10 in virus lysis buffer (0.1% SDS, 10 mM Tris/HCl pH 7.4, 1 mM EDTA) and incubated for 10 min at 56°C to release the viral genomes and the OD_260_ was determined. Calculations were based on 1 OD_260_ = 1.1×10^12^ particles/ml [51] .

Lentiviral vector production for shRNA encoding vectors was done by the service platform for lentiviral vector production of the Institute Federative de Recherche 66 of the Bordeaux 2 University. Prevalidated lentiviral vectors encoding shRNAs in the vector backbone pLKO.1 against Nedd4.1 and Nedd4.2 were purchased from the Mission™ shRNA collection from Sigma. For downregulation of Nedd4.1 we used NM_006154.1-1753s1c1 (sh4.1 (1), CCGGCCGGAGAATTATGGGTGTCAACTCGAGTTGACACCCATAATTCTCCGGTTTTT) against the coding sequence and NM_006154.1-3522s1c1 (sh4.1 (2), CCGGGCCTTTCTCTTGCCTGCATATCTCGAGATATGCAGGCAAGAGAAAGGCTTTTT) against the 3′ UTR. For downregulation of Nedd4.2 we used NM_015277.x-2772s1c1 (sh4.2 (1), CCGGGCGAGTACCTATGAATGGATTCTCGAGAATCCATTCATAGGTACTCGCTTTTT) against the coding sequence and NM_015277.x-3959s1c1 (sh4.2 (2), CCGGCCTGTTTGTATGCGTTTGCTACTCGAGTAGCAAACGCATACAAACAGGTTTTT) against the 3′ UTR. Control vectors for shRNAs encoded for shRNA against luciferase (Sigma) and control vectors for transduction and estimation of the titer encoded for GFP.

### Plasmids, siRNA

All sequences for protein VI were derived from Ad serotype 5 (Ad5) and cloned into the Gateway™ compatible entry vector pDONR221. Sequence verified DONR plasmids were used for recombination into Gateway™ compatible destination vectors for N-terminal fusion of mRFP (L30-mRFP, kindly provided by E. Bertrand). Bacterial expression vectors for protein VI are based on pET15b. Site-directed mutagenesis was used to change amino acids 148-PPSY-151 to 148-PGAA-151 in protein VI. N-terminal tagged expression vectors for Nedd4.1, and Nedd4.2 were provided by E. Bertrand [52] and tagged expression vectors for AIP4/Itch and WWP1 were a kind gift of Paul Bieniasz (Rockefeller University, New York). Bacterial expression vectors for catalytically active murine GST-Nedd4.2 and the inactive GST-Nedd4.2-DN was kindly provided by S. Kumar [53].

siRNAs were purchased as duplexes from EuroGentec (only the reverse strand is shown): Scramble (5′-CGCAAUUCGAUGUCCCGUGdTdT), Nedd4.1 (5′-AAACAACCCAGCCAGGCUCdTdA), Nedd4.2 (5′-CUGUGACUUUGUGUUGUGGdTdA), were previously described by Segura-Morales et al. (2005), AIP4/Itch siRNAs (5′-UCAUCAUUCUGAGAAGCACdTdT, [54] , and WWP1 siRNA (5′-CUUCUACGAUCAUCAACUCdTdT) was previously described by Chen et al. (2005). The WWP2 siRNA was a Smartpool from Dharmacon.

### RNA interference, adenoviral transduction and FACS analysis

Depletions were performed in 12-well dishes using 2×10^5^ U2OS cells. Cells were transfected after 24 and 48 h with 20 pmol of each siRNA duplex per well. Forty-eight hours after the first transfection cells were transduced using 30 physical particles per cell of Ad5-GFP virus for 3 h without prior pre-adsorption. Cells were harvested 24 h later and analyzed by flow cytometry for GFP expression and further processed for western blot analysis to verify knock-down efficiency. Acquisitions were done with FACSCalibur® or FACSCantoII® cytometer (BD Biosciences) and the data were processed and analyzed by the CellQuest® Pro and FACSDIVA® software (BD Biosciences).

### Labeling and infection assays

Purified Ad particles were labeled using the Alexa-488 microscale protein labeling kit (Invitrogen) using the manufacturers protocol. Infectivity of labeled virus preparations was determined by quantification of GFP-transduction. Only preparations with >90% activity where used. For time course experiments, U2OS cells were seeded at semiconfluency on coverslips. Pre-binding was done with 5000 physical particles per cell in 100 µl at 4°C on a shaking platform for 1 h. At t_0_ coverslips where rinsed in cold DMEM and transferred to pre-equilibrated (37°C, 5% CO_2_) DMEM. At indicated time points the cells where fixed and processed for IF analysis.

### Analysis of membrane penetration (Sarcin assay)

A549 lung epithelial cells were plated in 96-well plates at a density of 10,000 cells/well on the day before infection. The cells were washed once with DMEM without cysteine or methionine and supplemented, 2 mM glutamine, 10% dialyzed FCS, penicillin and streptomycin (DMEM-SA) and infected with the respective viruses in 50 µl DMEM-SA containing 0.1 mg/well α-sarcin (Sigma). The infected cells were incubated 30 min at 4°C to facilitate virus attachment and 90 min at 37°C to facilitate virus internalization. After this 50 µl of DMEM-SA containing 0.1 µCi of [35S]L-methionine (Hartmann Analytic) was added to each well and the cells were incubated for an additional 60 min at 37°C for labeling. The cells were then washed with 100 µl PBS and extracted in 150 µl lyses buffer containing 1% Triton-X100, 150 mM NaCl, 10 mM MgCl_2_, 20 mM Tris-HCl (pH 7.5) supplemented with 1× Complete™ protease inhibitor cocktail (Roche). The lysates were clarified by centrifugation at 20,000 g for 12 min. To remove the residual [35S]L-methionine, 100 µl cleared lysates were further purified with Zeba Desalt Spin Columns (Pierce). The incorporation [35S]L-methionine into the extracted fraction of newly synthesized proteins was measured by liquid scintillation using TRI-CARB 1900CA counter (Packard).

### Immunofluorescence (IF)

Cells grown on coverslips were rinsed in PBS and fixed with 4% PFA in PBS and blocked/permeabilized with IF-buffer (10% FCS in PBS and 0.1% Saponin). Primary and secondary antibodies where applied to the coverslip in IF-buffer for 1 h each. Cells were mounted in DAKO mounting media containing DAPI and analyzed by confocal- or epifluorescence microscopy. For IF involving microtubule staining cells were treated with crosslinkers prior to fixation. The following primary antibodies were used in this study: mouse anti-AcTubulin (kind gift from C. Janke, Montpellier), Mouse anti E2A (kind gift of T. Dobner, Hamburg), rabbit anti pericentrin (Abcam) and rabbit anti-protein VI antibodies raised against recombinant protein VI and affinity purified (see supplemental material). Secondary antibodies Alexa546 anti mouse was from Affinity Research and Atto647 anti rabbit was from Sigma.

### Microscopy and image analysis

Confocal pictures were taken on a Zeiss LSM 510 Meta confocal microscope or a Leica SP5 confocal microscope and epifluorescence pictures were taken on a Zeiss Axiolmager Z1 microscope with CoolSnap HQ Photometrics camera both equipped with Metamorph™ software. Confocal stacks where taken every 0.5 µm with a pinhole setting of 1 for all channels to achieve high local resolution. Images were processed using ImageJ and Adobe Photoshop™. Counting of viral particles was performed using the semi-automated cell counting tool from ImageJ. *Colocalization analysis:* Stacks from confocal images where combined as Z-projection using maximum intensity, converted into 8-bit images for each channel. Colocalization between protein VI and Ad was then determined using the colocalization finder plugin from ImageJ. *Live-Cell Imaging:* U2OS cells were seeded in 3.5cm glass-bottom dishes and transfected with 1.5 µg of protein VI-expressing vectors. Twenty-four h later the medium was replaced by pre-warmed OPTI-MEM (Gibco). Movies were acquired on a Nikon TE 2000 microscope with Cascade 512B 2 camera using Metamorph™ software for data acquisition (120 frames, 1 frame/sec). In some cases, 2 h before the acquisition, cells were incubated with either Nocodazole (Sigma) or Cytochalasin B (Sigma) diluted respectively at 0.4 µg/ml and 5 µg/ml in OPTI-MEM. Drugs were not removed during acquisition. Acquired movies were further processed using ImageJ to enhance protein VI particle detection by background subtraction and bleach correction. Particles were tracked using the spot-tracking tool of Imaris™ software to determine the length of their trajectories and the speed of their movement. Particle detection size was scaled to 0.75 µm and tracks were built with a maximum displacement of 1.5 µm between consecutive frames, a maximum gap size of 3 frames and a minimal track length of 20 s. At least 5 cells were analyzed for each condition that equals a minimum of 300 analyzed tracks per condition.

### Transmission electron microscopy

Three microliter of purified sample virus was adsorbed to a carbon-coated film (200 mesh grids). The grids with adsorbed virus were floated onto a solution of the negative stain (1% solution of uranyl acetate). The film was picked up by a copper EM grid and then air-dried. Specimens were examined under a HITACHI H7650 electron microscope operating at 80 kV and images were further processed using ImageJ software.

### Western blot and antibodies

Affinity purified rabbit anti-protein VI antibodies were used at a dilution of 1∶2000. Other antibodies used for the study were: rabbit polyclonal anti-Nedd4.1 and anti-Nedd4.2 antibodies that were a kind gift of O. Staub (Lausanne, Switzerland) (dilution 1∶1000), rabbit polyclonal anti-WWP2 antibody (sc-30052, Santa Cruz Biotechnology) (dilution 1∶200), goat polyclonal anti-WWP1 antibody (sc-11893, Santa Cruz Biotechnology) (dilution 1∶200), mouse monoclonal anti-AIP4/Itch antibody (sc-28367, Santa Cruz Biotechnology) (dilution 1∶100) and mouse monoclonal anti-GFP antibody (Roche) (dilution 1∶500). SDS-PAGE was done using 12% poly-acrylamide gels and transferred to nitrocellulose membranes. Membranes were blocked in TBS containing 10% of dry-milk and 0.01% of Tween 20 (Sigma), followed by over-night detection of antigens using primary antibodies diluted in TBS containing 10% of dry-milk and 0.01% of Tween 20 (Sigma). Primary antibodies were detected using HRP-conjugated secondary antibodies against rabbit, goat or mouse (Sigma) at a dilution of 1∶5000. Specific signals were revealed using the enhanced chemiluminescence detection system (ECL) (PerkinElmer).

### Statistical analysis

Data are presented as mean, error bars as STD. Statistical analysis if not indicated otherwise was done using unpaired students t-test (*:P<0.05; **:P<0.01; ***:P<0.005).

## Supporting Information

Figure S1 Protein VI release in U2OS cells during Ad entry. Ad5-VI-wt-488 was pre-bound to cells at 4°C (top row) and shifted to 37°C for 5min (second row), 15min (third row) and 45min (bottom row). Protein VI was detected using affinity purified anti-protein VI antibodies (left column) and Ad by detecting the Alexa-488 fluorescent signal (middle column). A composite of both signals including the nucleus (in greyscale) is shown in the left column. The inset shows a magnification of representative virus and protein VI signals in the small white box. Protein VI signals are shown in red, Ad is shown in green and colocalization of protein VI and Ad is shown in yellow. The scale bar is 10 µm. The rabbit polyclonal serum against protein VI was generated against recombinant purified His-tagged protein VI. Rabbit serum that reacted positive and specific against protein VI in western blots of purified viruses was used for further affinity purification for use in immunofluorescence applications. Affinity purification was done using recombinant purified protein VI coupled to CnBr^+^-activated sepharose beads. Bound antibodies were eluted with 0.1 M glycin ph2, neutralized with 2M Tris pH 8.8 and dialyzed against PBS. Affinity purified antibodies were used at 1∶250 dilutions in immunofluorescence.
(2.69 MB TIF)

Figure S2 Alignment of the PPxY motif in the sequence of protein VI from different human and non-human adenovirus serotypes. A partial alignment of protein VI sequences from different human adenoviral serotypes as well as non-human adenoviruses from the genus *Mastadenovirus* is shown. The conserved ubiquitin ligase-recruiting motif is boxed. Sequences were retrieved from public databases with the following accession numbers; canine (CAV-2, AP_000621), bovine (boAd3, AP_000031), huAd3 (serotype B, ABB17802), huAd35 (serotype B, AP_000584), huAd4 (serotype E, YP_068031), huAd17 (serotype D, AP_000149), huAd2 (serotype C, AP_000174), huAd5 (serotype C, AP_000210), huAd12 (serotype A, AP 000120), huAd40 (serotype F, NP_040861), murine (muAd1, AP_000350).
(0.45 MB TIF)

Figure S3 Construction of mutant Ad5 with altered PPxY motif in protein VI using BAC technology. A) To construct a bacterial artificial chromosome (BAC) carrying an infectious Ad5 genome, we cloned an AdEasy system (Stratagene) based virus genome into pKSB2 vector as described previously (Warming et al. [57]; Ruzsics et al. [34]). This recombinant Ad5 lacked the E1 and E3 regions and carried an FRT site in the place of its E1 region, which was introduced through an FRT containing pShuttle (Stratagene) clone. The resulting BAC, was termed pAd5-FRT and can be reconstituted to fully infectious recombinant Ad5 viruses after transfection of E1 complementing cell lines such as 293 cells. To construct protein VI-modified viruses, pAd5-FRT was transformed into the *E. coli* strain SW102, which encodes the λ-red recombination system from the bacteriophage under a heat-inducible promoter [57]. We next amplified a Kanamycin resistance cassette using primers with 50 nt 5′ extensions homologous to protein VI coding regions. The forward primer was flanked with a homology located upstream to the PPSY motif and introduced a ClaI site into the protein VI ORF without affecting its amino acid sequence. The homology region attached to the reverse primers carried the same ClaI site and overlapped with the PPSY motif. Two different reverse primers were generated: one carried an unaltered PPSY motif and another that encoded the amino acids PGAA instead of PPSY and introduced an additional Pst I site by the new coding sequence. The PCR products were transformed into the SW102 bacteria harbouring the Ad5-BAC following heatshock to induce red-recombination. Chloramphenicol and kanamycin double resistant clones were selected and BAC DNA was prepared from individual clones. The isolated BAC DNA was digested with ClaI and subsequently re-ligated. This procedure eliminated the kanamycin cassette and reconstituted the protein VI ORF concomitant to the re-circularisation of the ClaI treated BACs, because there was no other ClaI site present in the rest of the pAd5-FRT. If the reverse primer with an intact PPSY motif was used for amplification, the wild type protein VI amino acid sequence was reconstituted with a silent genetic tag introducing a ClaI site. If the reverse primer with PGAA motif was used for amplification, the protein VI coding sequence was modified at two positions i) a silent genetic tag was inserted introducing a ClaI site as above and ii) the PPSY motif was replaced by the PGAA motif introducing a new PstI site. After the ClaI treatment and re-ligation the modified genomes were retransformed in *E. coli* DH10B. Kanamycin negative colonies were identified by replica plating and the resulting mutants were analysed by restriction digestions and verified by sequencing. The mutant Ad genomes were released from the respective BACs by Pac I digestion and transfected into 293 cells. Following the appearance of cytopathic effects the virus was amplified and purified by double CsCl banding, dialyzed into PBS/10% Glycerol and snap frozen. B) To verify the identity of the purified virions and analyse whether detectable reversion occurred during reconstitution and propagation of the virus stocks viral DNA was extracted from purified virions and was PCR amplified with protein VI specific primers. The PCR products were digested with Pst I (left) and Cla I (right) to identify the recombinant viruses with or without the altered protein VI sequences. To insert a GFP expression cassette we used bacterial Flp-recombination using the FRT site in the E1-deleted region of the Ad5-VI-wt and Ad5-VI-M1 BACs. We cloned the left end of the Ad5 (nt 1–341) flanked by a Pac I restriction site into the plasmid pOriR6K-ie [55]; GenBank Acc. AY700022) upstream of its FRT site. We also replaced its zeocin resistance marker with an pGPS1.1(NEB) derived kanamycin cassette and cloned an EGFP ORF from pEGFP-N1 (Clontech) in its expression locus and termed this plasmid pO6-A5-gfp. The pOriR6Kie derived plasmids can only be maintained in special *E. coli* strains such as PIR1 (Invitrogen) because they are dependent on the presence of R6Kγ phage replicase [55]. To carry out the recombination, *E. coli* strain DH10B (Invitrogen) was co-transformed with pAd5-FRT derived BACs and pCP20 encoding the Flp-recombinase [56] and cultured at 30°C. The Flp recombinations were carried out as described in Bubeck et al. [55]. Briefly, the *E. coli* cell carrying the target BACs and the Flp expression plasmid pCP20 were transformed with the pO6-A5-GFP and selected for chloramphenicol and kanamycin resistance upon induction of the Flp expression by a temperature shift to 43°C. This treatment induced a recombination between the two FRT sites (one in the pAd5-FRT derivative and one on the pO6-A5-gfp) and induced unification of the BAC and the pO6 construct. Only the recombined construct survived the double selection because pO6 constructs are not maintained in DH10B. This approach essentially replaced the old left end of the Ad5 BAC by a CMV promoter driven GFP-expression cassette containing fragment, which also possessed all the *cis* elements needed for virus reconstitution as described above.
(0.45 MB TIF)

Figure S4 The PPxY-mutant Ad5GFP-VI-M1 forms smaller and fewer plaques. A) Shown is a comparison of the growth of individual plaques starting from a single infected cell. E1 complementing 911 cells were infected at low multiplicity of infection with Ad5GFP-VI-wt and the PPxY mutant virus Ad5GFP-VI-M1 for 24 h and then washed and overlayed with agarose. Virus growth was monitored by the appearance of GFP-positive cells and images of representative cells/plaques were taken on days 1, 3, 9 and 12. The images in the left row show the plaque formation of the wild type virus 1, 3, 9 and 12 days after the initial infection (top-to-bottom). At day 9 and 12 significant large plaques with lesions of the cell monolayer can be observed. In contrast the mutant virus to the right shows a slow expansion of GFP-positive plaques with less damage to the cell monolayer. Images are superimpositions of the GFP signal and the phase contrast image of the monolayer. B) The image shows the damage in the cell monolayer caused by plaque formation on day 12. The arrow indicates the average size of the plaque. The scale bar is 50 µm.
(2.12 MB TIF)

Figure S5 Penton is a target for ubiquitylation following partial disassembly of the virus. A) Localization and sequences of conserved PPxY-motifs in protein VI and penton for Ad5 (black box). Note that processed protein VI is shown as it is present in the capsid during viral entry. B) Schematic representation of the *in vitro* ubiquitylation assay. Virus disassembly was induced by mild heat-treatment, *in vitro* ubiquitylated using ubiquitin or recombinant GST-ubiquitin, recombinant E1 and E2, an energy regenerating system and purified cytosol as source for the E3-ligase and analyzed by western blot. Controls lack the mild heat-treatment. C) Western blot analysis of viral capsid proteins penton, fiber and protein IIIA following capsid disassembly and *in vitro* ubiquitylation. Heat-treatment is indicated above each lane. Antibodies are indicated to the right of each blot. Specific bands are labeled accordingly. Grey arrows indicate band shifts due to ubiquitylation, black arrows indicates the unmodified protein. D) Western blot analysis of *in vitro* ubiquitylation reactions of heat-treated viral particles. Heat treatment is indicated above each lane. For individual reactions the cytosol was depleted with recombinant fiber beads (control), recombinant VI-wt beads or recombinant VI-M1 beads as indicated above each lane. Reactions were blotted with anti-penton. The assay shows that the ubiquitylation activity can be depleted from cytosol with recombinant wt protein VI but not when the PPSY motif is mutated. The same assay also abolishes protein VI ubiquitylation showing that similar ubiquitylation activities are responsible (data not shown). E) Protein VI depleted cytosol renders Nedd4.2 active for penton ubiquitylation. *In vitro* ubiquitylation reactions using catalytically active or inactive Nedd4.2 substituted with cytosol depleted by protein VI-wt (as indicated above each lane) and analyzed by western blot with anti-penton antiserum. Black arrows indicate ubiquitylated proteins, grey arrows the unmodified protein. This assay shows that additional cytosolic factors are required for full Nedd4.2 activity for penton ubiquitylation.
(0.73 MB TIF)

Figure S6 Protein VI with altered PPxY motif does not colocalize with Nedd4 ligases. Protein VI with PPSY motif mutated to PGAA was N-terminally fused to mRFP and co-transfected with GFP-Nedd4.1 (top row), GFP-Nedd4.2 (second row), GFP-AIP4/Itch (third row), GFP-WWP1 (forth row) and GFP-WWP2 (bottom row). Confocal images of representative cells are shown and the mRFP signal for VI (left column), the GFP signal for the ligases (centre column) and the merged signals together with DAPI stain of the nucleus (right column) is indicated above each column. Transfected plasmids are indicated left of each row. Note that the cytoplasmic localization of each ligase is similar to that in cells without cotransfection of protein VI (data not shown).
(3.90 MB TIF)

Video S1 Intracellular dynamics of protein VI. U2OS cells were transfected with VI-wt, fused to mRFP. The movie was acquired using a Nikon TE 2000 microscope equipped with Cascade 512B 2 camera at 1 frame per second. The movie shows rapid intracellular movement of VI-wt depicting compartments resembling vesicular, tubulo-vesicular and reticular structures.
(0.62 MB MOV)

Video S2 Comparison of intracellular dynamics for VI-wt vs. VI-M1 vs. VI-wt (+ nocodazole). U2OS cells were transfected with VI-wt (left and right panel) or VI-M1 (middle panel) fused to mRFP. The cell to the right was treated with nocodazole (5 µg/ml) prior to image acquisition. Movies were acquired using a Nikon TE 2000 microscope equipped with Cascade 512B 2 camera at 1 frame per second. The movies were labeled and assembled using imageJ and converted into QuickTime™ movies. All three displayed movies were taking at the same frame rate. The movies show rapid intracellular movement of VI-wt at nearly real-time (left). VI-M1 shows strongly reduced movement (middle). Treatment of VI-wt transfected cells with nocodazole, strongly reduces the VI-wt movement resembling VI-M1 dynamics (right).
(1.21 MB MOV)
