# Supplementary figures and images for: A Capsid-Encoded PPxY-Motif Facilitates Adenovirus Entry
Source: PLoS Pathog. 2010 Mar 19;6(3):e1000808. doi: 10.1371/journal.ppat.1000808 (PMC2841620; doi:10.1371/journal.ppat.1000808)

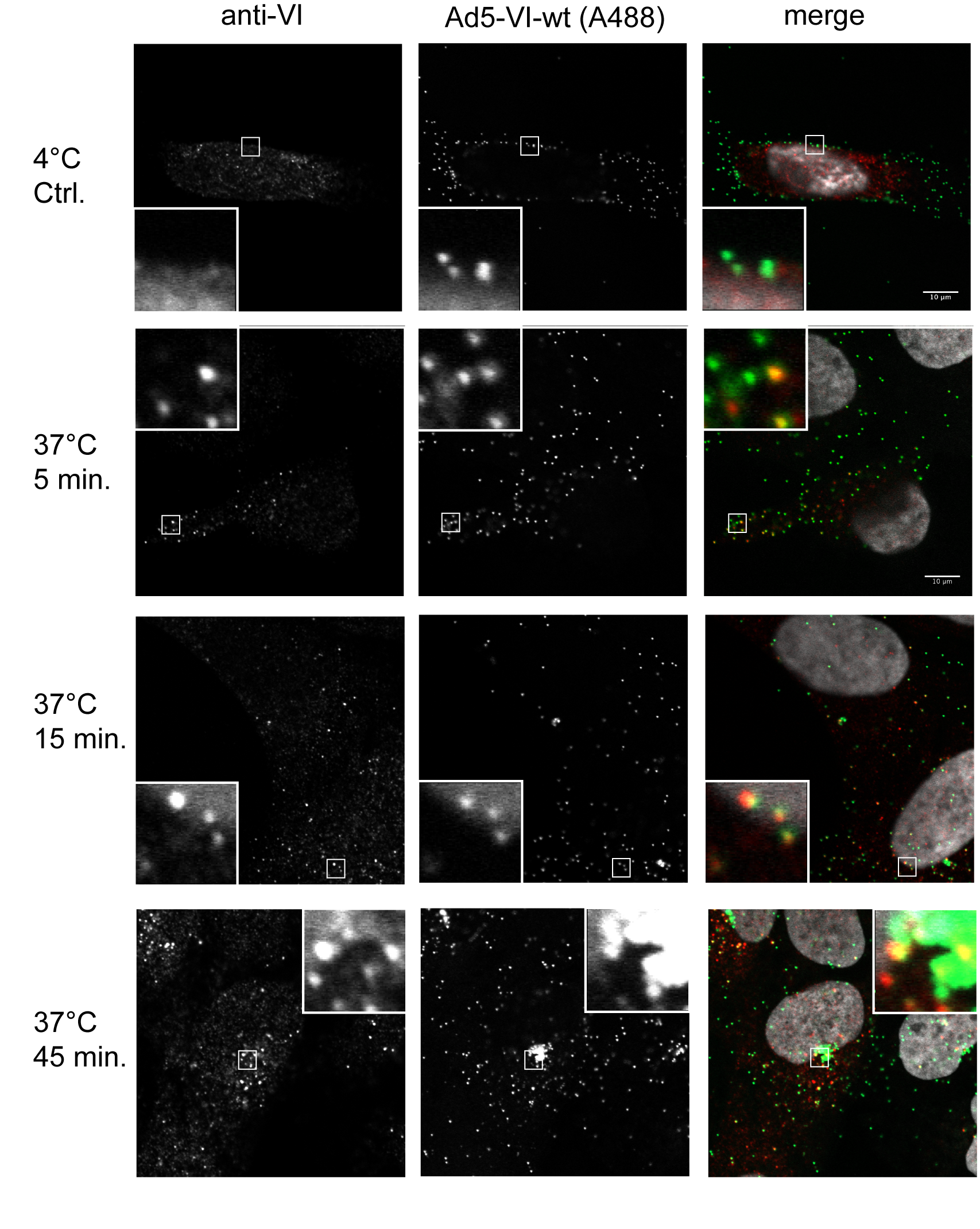

Supplement: Figure S1 — Protein VI release in U2OS cells during Ad entry. Ad5-VI-wt-488 was pre-bound to cells at 4°C (top row) and shifted to 37°C for 5min (second row), 15min (third row) and 45min (bottom row). Protein VI was detected using affinity purified anti-protein VI antibodies (left column) and Ad by detecting the Alexa-488 fluorescent signal (middle column). A composite of both signals including the nucleus (in greyscale) is shown in the left column. The inset shows a magnification of representative virus and protein VI signals in the small white box. Protein VI signals are shown in red, Ad is shown in green and colocalization of protein VI and Ad is shown in yellow. The scale bar is 10 µm. The rabbit polyclonal serum against protein VI was generated against recombinant purified His-tagged protein VI. Rabbit serum that reacted positive and specific against protein VI in western blots of purified viruses was used for further affinity purification for use in immunofluorescence applications. Affinity purification was done using recombinant purified protein VI coupled to CnBr+-activated sepharose beads. Bound antibodies were eluted with 0.1 M glycin ph2, neutralized with 2M Tris pH 8.8 and dialyzed against PBS. Affinity purified antibodies were used at 1∶250 dilutions in immunofluorescence. (2.69 MB TIF) [file ppat.1000808.s001.tif]

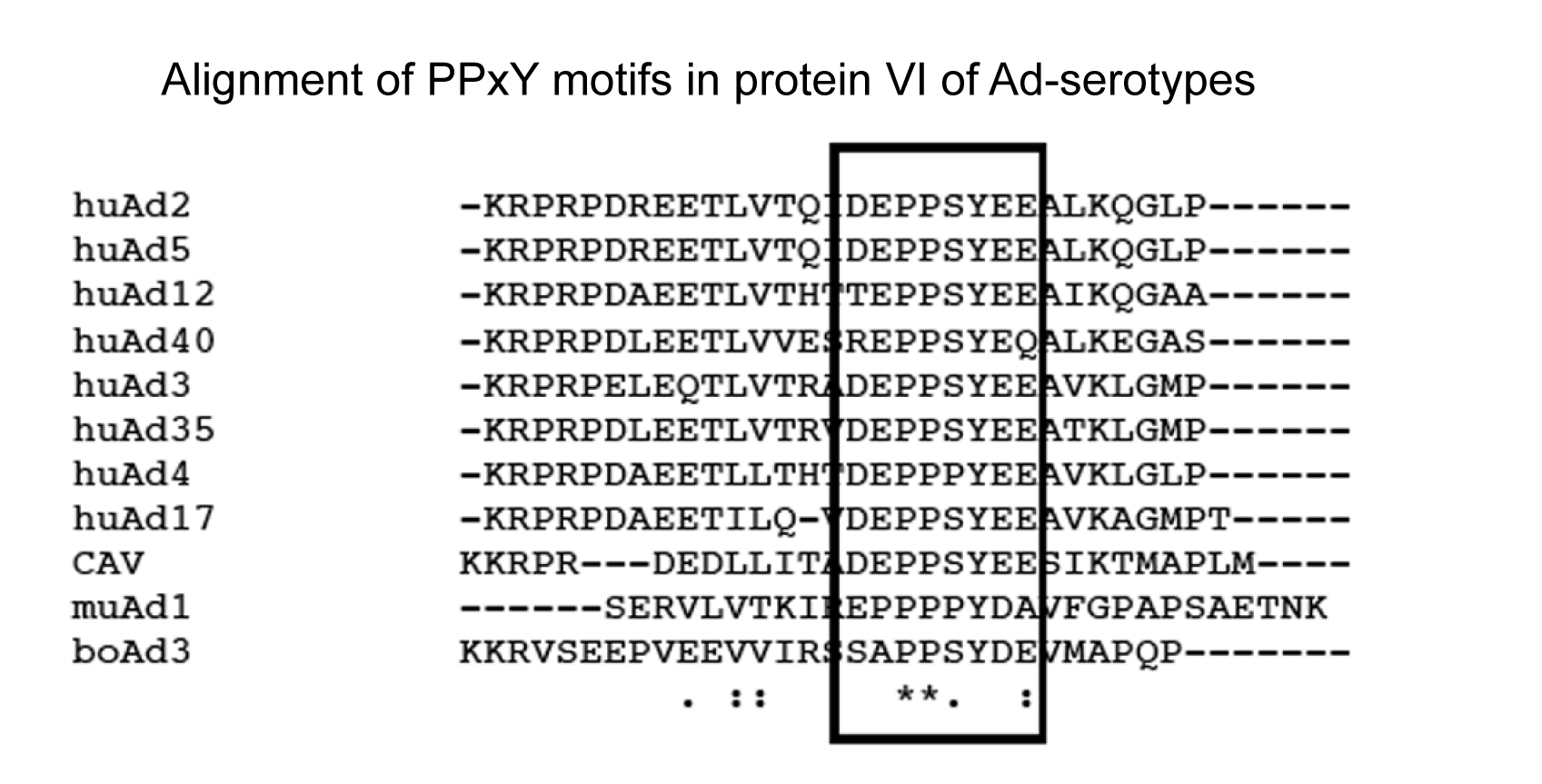

Supplement: Figure S2 — Alignment of the PPxY motif in the sequence of protein VI from different human and non-human adenovirus serotypes. A partial alignment of protein VI sequences from different human adenoviral serotypes as well as non-human adenoviruses from the genus Mastadenovirus is shown. The conserved ubiquitin ligase-recruiting motif is boxed. Sequences were retrieved from public databases with the following accession numbers; canine (CAV-2, AP_000621), bovine (boAd3, AP_000031), huAd3 (serotype B, ABB17802), huAd35 (serotype B, AP_000584), huAd4 (serotype E, YP_068031), huAd17 (serotype D, AP_000149), huAd2 (serotype C, AP_000174), huAd5 (serotype C, AP_000210), huAd12 (serotype A, AP 000120), huAd40 (serotype F, NP_040861), murine (muAd1, AP_000350). (0.45 MB TIF) [file ppat.1000808.s002.tif]

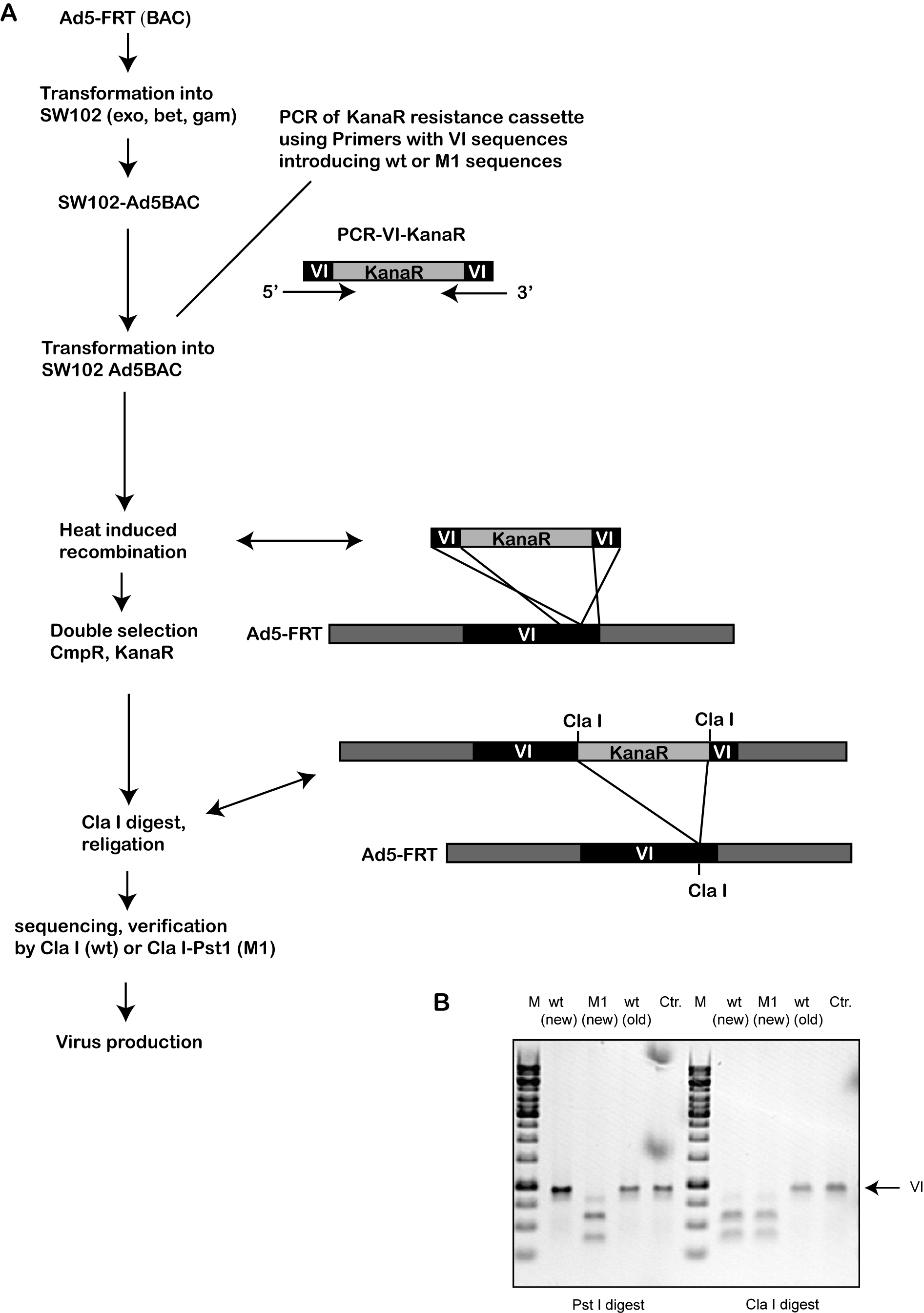

Supplement: Figure S3 — Construction of mutant Ad5 with altered PPxY motif in protein VI using BAC technology. A) To construct a bacterial artificial chromosome (BAC) carrying an infectious Ad5 genome, we cloned an AdEasy system (Stratagene) based virus genome into pKSB2 vector as described previously (Warming et al. [57]; Ruzsics et al. [34]). This recombinant Ad5 lacked the E1 and E3 regions and carried an FRT site in the place of its E1 region, which was introduced through an FRT containing pShuttle (Stratagene) clone. The resulting BAC, was termed pAd5-FRT and can be reconstituted to fully infectious recombinant Ad5 viruses after transfection of E1 complementing cell lines such as 293 cells. To construct protein VI-modified viruses, pAd5-FRT was transformed into the E. coli strain SW102, which encodes the λ-red recombination system from the bacteriophage under a heat-inducible promoter [57]. We next amplified a Kanamycin resistance cassette using primers with 50 nt 5′ extensions homologous to protein VI coding regions. The forward primer was flanked with a homology located upstream to the PPSY motif and introduced a ClaI site into the protein VI ORF without affecting its amino acid sequence. The homology region attached to the reverse primers carried the same ClaI site and overlapped with the PPSY motif. Two different reverse primers were generated: one carried an unaltered PPSY motif and another that encoded the amino acids PGAA instead of PPSY and introduced an additional Pst I site by the new coding sequence. The PCR products were transformed into the SW102 bacteria harbouring the Ad5-BAC following heatshock to induce red-recombination. Chloramphenicol and kanamycin double resistant clones were selected and BAC DNA was prepared from individual clones. The isolated BAC DNA was digested with ClaI and subsequently re-ligated. This procedure eliminated the kanamycin cassette and reconstituted the protein VI ORF concomitant to the re-circularisation of the ClaI treated BACs, because ther [file ppat.1000808.s003.tif]

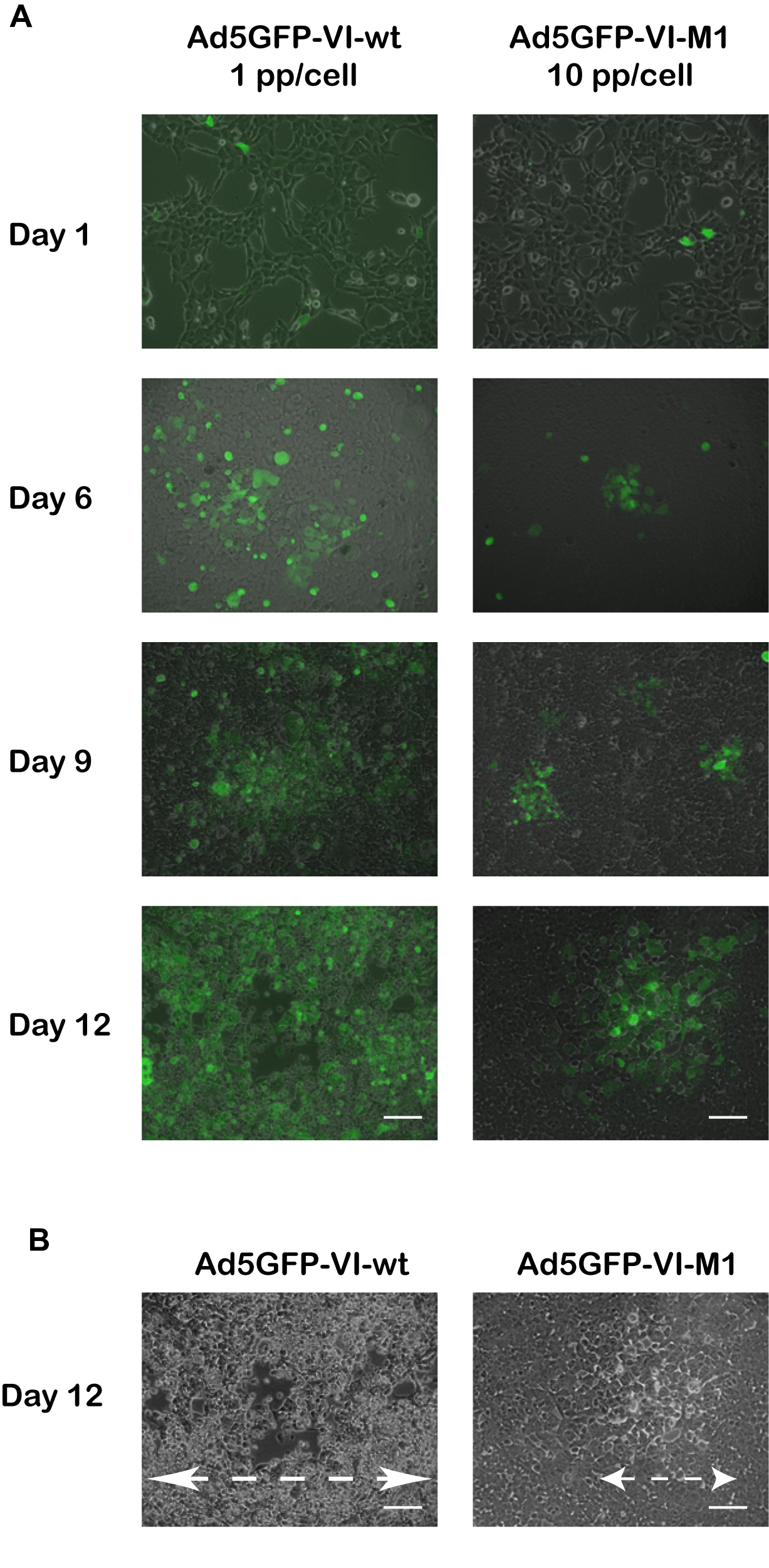

Supplement: Figure S4 — The PPxY-mutant Ad5GFP-VI-M1 forms smaller and fewer plaques. A) Shown is a comparison of the growth of individual plaques starting from a single infected cell. E1 complementing 911 cells were infected at low multiplicity of infection with Ad5GFP-VI-wt and the PPxY mutant virus Ad5GFP-VI-M1 for 24 h and then washed and overlayed with agarose. Virus growth was monitored by the appearance of GFP-positive cells and images of representative cells/plaques were taken on days 1, 3, 9 and 12. The images in the left row show the plaque formation of the wild type virus 1, 3, 9 and 12 days after the initial infection (top-to-bottom). At day 9 and 12 significant large plaques with lesions of the cell monolayer can be observed. In contrast the mutant virus to the right shows a slow expansion of GFP-positive plaques with less damage to the cell monolayer. Images are superimpositions of the GFP signal and the phase contrast image of the monolayer. B) The image shows the damage in the cell monolayer caused by plaque formation on day 12. The arrow indicates the average size of the plaque. The scale bar is 50 µm. (2.12 MB TIF) [file ppat.1000808.s004.tif]

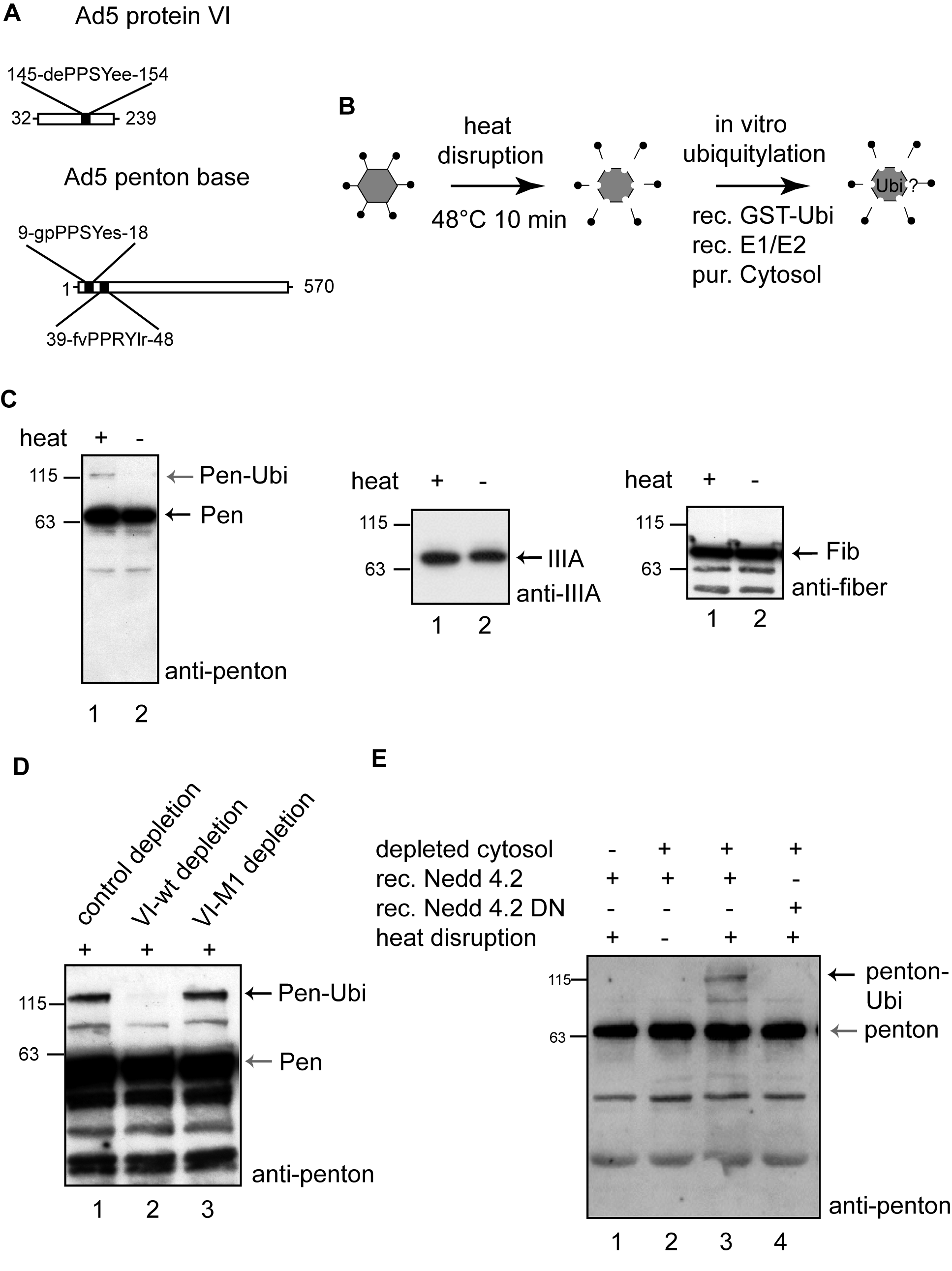

Supplement: Figure S5 — Penton is a target for ubiquitylation following partial disassembly of the virus. A) Localization and sequences of conserved PPxY-motifs in protein VI and penton for Ad5 (black box). Note that processed protein VI is shown as it is present in the capsid during viral entry. B) Schematic representation of the in vitro ubiquitylation assay. Virus disassembly was induced by mild heat-treatment, in vitro ubiquitylated using ubiquitin or recombinant GST-ubiquitin, recombinant E1 and E2, an energy regenerating system and purified cytosol as source for the E3-ligase and analyzed by western blot. Controls lack the mild heat-treatment. C) Western blot analysis of viral capsid proteins penton, fiber and protein IIIA following capsid disassembly and in vitro ubiquitylation. Heat-treatment is indicated above each lane. Antibodies are indicated to the right of each blot. Specific bands are labeled accordingly. Grey arrows indicate band shifts due to ubiquitylation, black arrows indicates the unmodified protein. D) Western blot analysis of in vitro ubiquitylation reactions of heat-treated viral particles. Heat treatment is indicated above each lane. For individual reactions the cytosol was depleted with recombinant fiber beads (control), recombinant VI-wt beads or recombinant VI-M1 beads as indicated above each lane. Reactions were blotted with anti-penton. The assay shows that the ubiquitylation activity can be depleted from cytosol with recombinant wt protein VI but not when the PPSY motif is mutated. The same assay also abolishes protein VI ubiquitylation showing that similar ubiquitylation activities are responsible (data not shown). E) Protein VI depleted cytosol renders Nedd4.2 active for penton ubiquitylation. In vitro ubiquitylation reactions using catalytically active or inactive Nedd4.2 substituted with cytosol depleted by protein VI-wt (as indicated above each lane) and analyzed by western blot with anti-penton antiserum. Black arrows indicate ubiquitylated proteins, gr [file ppat.1000808.s005.tif]

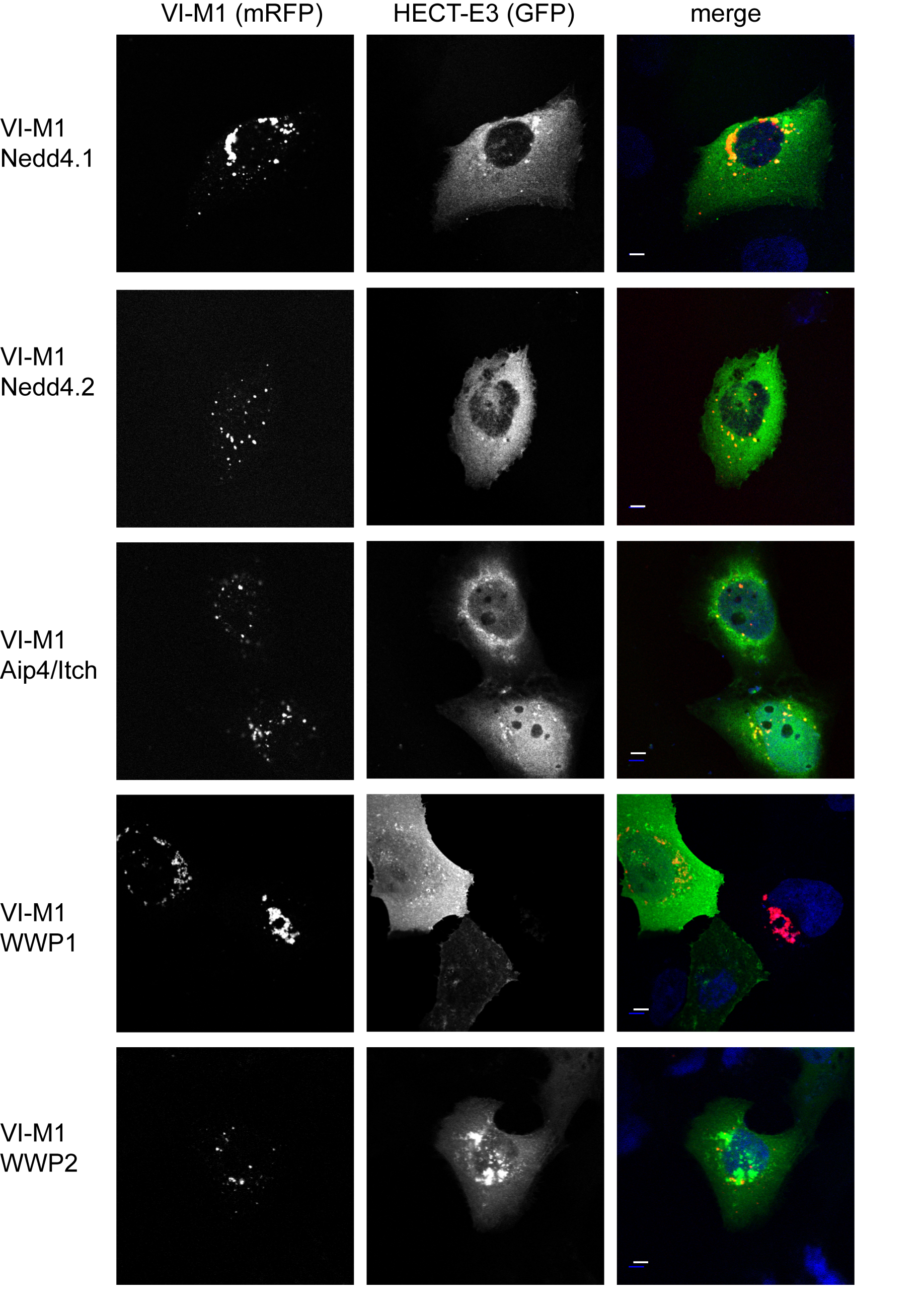

Supplement: Figure S6 — Protein VI with altered PPxY motif does not colocalize with Nedd4 ligases. Protein VI with PPSY motif mutated to PGAA was N-terminally fused to mRFP and co-transfected with GFP-Nedd4.1 (top row), GFP-Nedd4.2 (second row), GFP-AIP4/Itch (third row), GFP-WWP1 (forth row) and GFP-WWP2 (bottom row). Confocal images of representative cells are shown and the mRFP signal for VI (left column), the GFP signal for the ligases (centre column) and the merged signals together with DAPI stain of the nucleus (right column) is indicated above each column. Transfected plasmids are indicated left of each row. Note that the cytoplasmic localization of each ligase is similar to that in cells without cotransfection of protein VI (data not shown). (3.90 MB TIF) [file ppat.1000808.s006.tif]
